# Regulation and expression of sexual differentiation factors in embryonic and extragonadal tissues of Atlantic salmon

**DOI:** 10.1186/1471-2164-12-31

**Published:** 2011-01-13

**Authors:** Kristian R von Schalburg, Motoshige Yasuike, Ryosuke Yazawa, Johan G de Boer, Linda Reid, Stacy So, Adrienne Robb, Eric B Rondeau, Ruth B Phillips, William S Davidson, Ben F Koop

**Affiliations:** 1 Department of Biology, Centre for Biomedical Research, University of Victoria, Victoria, British Columbia, V8W 3N5, Canada; 2 Tokyo University of Marine Science and Technology, Tateyama, Chiba, 294-0308, Japan; 3 Biological Sciences, Washington State University, Vancouver, Washington, 98686-9600, USA; 4 Department of Molecular Biology and Biochemistry, Simon Fraser University, Burnaby, British Columbia, V5A 1S6, Canada

## Abstract

**Background:**

The products of *cyp19*, *dax*, *foxl2*, *mis*, *sf1 *and *sox9 *have each been associated with sex-determining processes among vertebrates. We provide evidence for expression of these regulators very early in salmonid development and in tissues outside of the hypothalamic-pituitary-adrenal/gonadal (HPAG) axis. Although the function of these factors in sexual differentiation have been defined, their roles in early development before sexual fate decisions and in tissues beyond the brain or gonad are essentially unknown.

**Results:**

Bacterial artificial chromosomes containing salmon *dax1 *and *dax2*, *foxl2b *and *mis *were isolated and the regulatory regions that control their expression were characterized. Transposon integrations are implicated in the shaping of the *dax *and *foxl2 *loci. Splice variants for *cyp19b1 *and *mis *in both embryonic and adult tissues were detected and characterized. We found that *cyp19b1 *transcripts are generated that contain 5'-untranslated regions of different lengths due to cryptic splicing of the 3'-end of intron 1. We also demonstrate that salmon *mis *transcripts can encode prodomain products that present different C-termini and terminate before translation of the MIS hormone. Regulatory differences in the expression of two distinct aromatases *cyp19a *and *cyp19b1 *are exerted, despite transcription of their transactivators (ie; *dax1*, *foxl2*, *sf1*) occurring much earlier during embryonic development.

**Conclusions:**

We report the embryonic and extragonadal expression of *dax*, *foxl2*, *mis *and other differentiation factors that indicate that they have functions that are more general and not restricted to steroidogenesis and gonadogenesis. Spliced *cyp19b1 *and *mis *transcripts are generated that may provide regulatory controls for tissue- or development-specific activities. Selection of *cyp19b1 *transcripts may be regulated by DAX-1, FOXL2 and SF-1 complexes that bind motifs in intron 1, or by signals within exon 2 that recruit splicing factors, or both. The potential translation of proteins bearing only the N-terminal MIS prodomain may modulate the functions of other TGF β family members in different tissues. The expression patterns of *dax1 *early in salmon embryogenesis implicate its role as a lineage determination factor. Other roles for these factors during embryogenesis and outside the HPAG axis are discussed.

## Background

Sex determination and early gonadal development depends upon specific embryonic gene programs that activate differentiation of the bipotential primodium. These processes hinge upon the expression of both sex-linked and autosomal genes that promote or antagonize male or female cell fate pathways. The products of some genes are essential for female (WNT-4 (Wingless-related MMTV integration site-4) and follistatin) [1] and male (SRY (Sex-determining region of the Y chromosome) and FGF-9 (fibroblast growth factor-9)) [2] sex-determination in mammals. In mammals, as well as teleosts, DAX-1 (Dosage-sensitive sex reversal, adrenal hypoplasia congenital, critical region on the X-chromosome, gene-1), FOXL2 (forkhead box L2), cytochrome P450 aromatase, MIS (Mullerian inhibiting substance), SF-1 (steroidogenic factor-1), SOX-9 (SRY-related, high-mobility group (HMG) box (SRY box)-9), WT-1 (Wilm's tumour-1) [3-6] and many other gene products contribute in directing sex-determined fates, as well as to subsequent gonad development and function [7-9 and refs. therein].

SF-1 is central to the activity of many of these genes as well as a large component of steroidogenic genes. Both SF-1 and DAX-1 are orphan receptors for which no ligand has yet been unequivocally identified, but which do possess ligand-binding domains [10]. SF-1 and DAX-1 interact cooperatively or antagonistically to influence various target gene activities [10]. DAX-1 modulates transcription of genes such as aromatase [11] and MIS [12] through direct interactions with SF-1 and the recruitment of other factors. Functional interactions of SF-1 with WT-1 or DAX-1 promotes or inhibits the expression of MIS [13]. The products of *dax1*, *foxl2 *and *sf1 *each play critical roles in the transcriptional regulation of aromatase (*cyp19*) genes [6,11,14-17].

Elucidation of the factors that promote or repress the transcription of these differentiation factors will also help to characterize the regulatory programs they initiate. Conservation of transcription factor binding motifs in the promoters of mammalian and teleost genes point to shared regulatory regimes. The expression patterns of the genes described here may be deciphered in part by examination of their promoter regions. Antagonism between DAX-1 and FOXL2 or SF-1 and SOX factors may be common themes for activation or repression among many of these differentiation factors [11,18-23].

Although our understanding of the action of various sex determination and differentiation factors in early gonad development is well-established, our knowledge of other roles that these regulators may play before formation of sexual tissues is limited. Sex-specific regulation of various steroidogenic and differentiation regulators in trout have been demonstrated by molecular studies to occur about one month before observable morphological differences [5]. Histologically discernable features of gonad differentiation in salmonids become manifest approximately five weeks after hatching [5,14].

In the Atlantic salmon (*Salmo salar*), we have detected the expression of *dax1/2*, *foxl2a/b *and *mis *(and *sf1 *and *sox9 *factors) more than three weeks before even the hatching stage. In this paper we show that the genes we examined were expressed several months before morphologically discernable differentiation of the gonad. This is the first time, other than for *dax1*, that this has been demonstrated for the genes we selected. We also demonstrate broad expression of these regulators in various extragonadal adult tissues. Therefore genes previously considered to be involved in gonadogenesis and steroidogenesis are apparently more general in their transcriptional programming, at least in salmon.

Furthermore, we have isolated, sequenced and assembled BACs containing these genes and analyzed their proximal promoters. Our expression studies also reveal unique splicing activities that occur among some of these regulators. Taken together, we suggest that the roles of *dax1/2*, *foxl2a/b*, *mis *and *sf1 *are not confined to differentiation and development of gonadal (and brain) cell lineages and examine the potential influence of the novel splicing and transcriptional regulatory mechanisms of these factors within and outside the HPAG axis.

## Results

### Stages that present challenges during development

**Figure 1:**
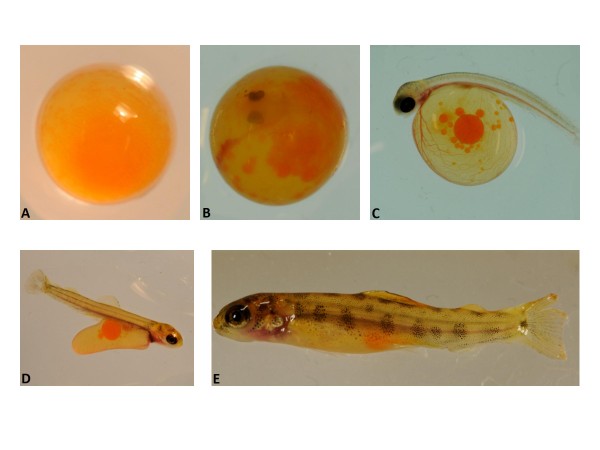
**Images of selected development stages**. Ages based on day ((D) post-fertilization. A. D7. B. D23. C. D38. D. D53. E. D71.

To characterize the stages of development of the Atlantic salmon and identify potentially problematic stages, we assayed mortality rates during embryonic and larval development. The day of fertilization was Day (D) 0, and hatching and yolk sac absorption occurred between D38 to D40 and D68 to D70, respectively. Images of selected stages taken during the course of early development are presented in Figure 1. We counted the mortalities each collection day during the development study. We found that there were at least four stages which can present potential developmental challenges (see Additional file 1). One stage comes at D13.5, the point at which we noted eye formation was initiated. Next, from D38 to D50 is a period when hatching had begun. Most larvae hatched between D38 and D40, but there also were a large number for which hatching was delayed. The large peak at D49 (see Additional file 1) marks 184 mortalities due to late, incomplete or unsuccessful hatching. Many larvae in this group failed to complete hatching or had survival problems once hatched. The two small peaks that follow this period represent morphological abnormalities that arose during alevin development (ie; curved backs).

### Early expression of sex differentiation factors

**Figure 2:**
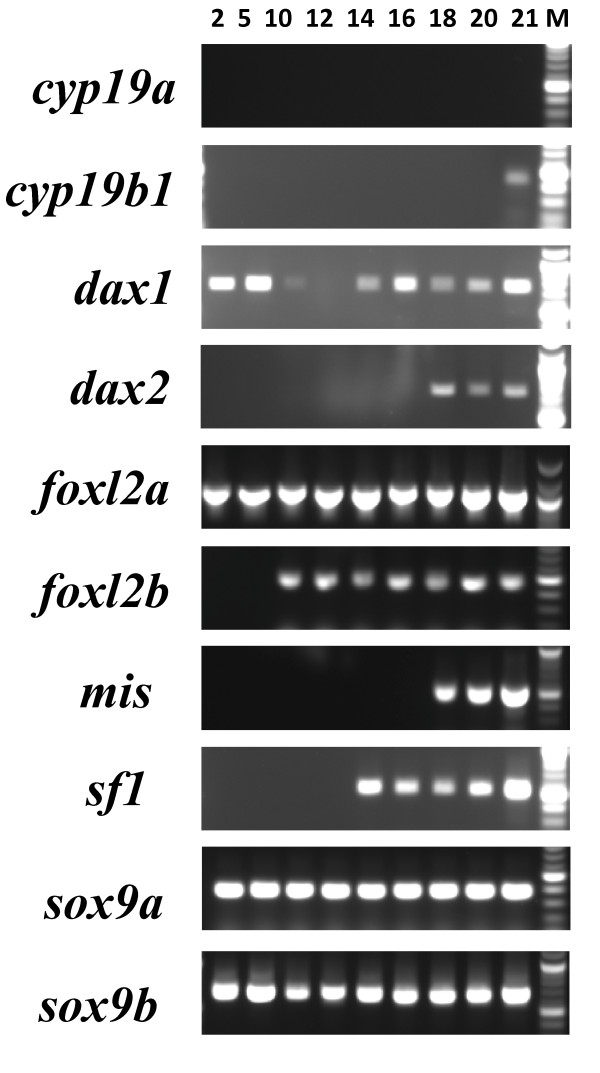
**Reverse-transcriptase PCR validation of gene-specific cDNA expression during embryonic stages of development**. Development stage indicated by day (Days 2 through 21) post-fertilization. For determining the lengths of each amplicon, the strongest marker bands indicate fragment lengths of 500 bp (*cyp19b1*, *foxl2b*, *mis*, *sf1*, *sox9a *and *sox9b*) or 1000 bp (*dax1*, *dax2 *and *foxl2a*).

It became increasingly clear from preliminary studies that the expression of known sex differentiation regulators were expressed much earlier in salmonid development than we had expected. To determine when expression of these sex differentiation factors was initiated, we prepared RNA from embryos two days post-fertilization (D2) and succeeding time points. Our RT-PCR results show that the mRNA for many of these genes is present very early in development (Figure 2), at least one month ahead of previously characterized expression of regulators linked to differentiation of the salmonid gonad [5,14]. It was necessary to reamplify the early amplification products of *dax2*, *foxl2a*, *foxl2b*, *mis *and *sf1 *for presentation in Figure 2. We do not know if these transcripts are maternally contributed or if they are the result of zygotic transcription. To the best of our knowledge, for the genes we detected at these early stages, there presently is only evidence for the expression of *dax1 *in embryonic cells in mammals [24].

**Figure 3:**
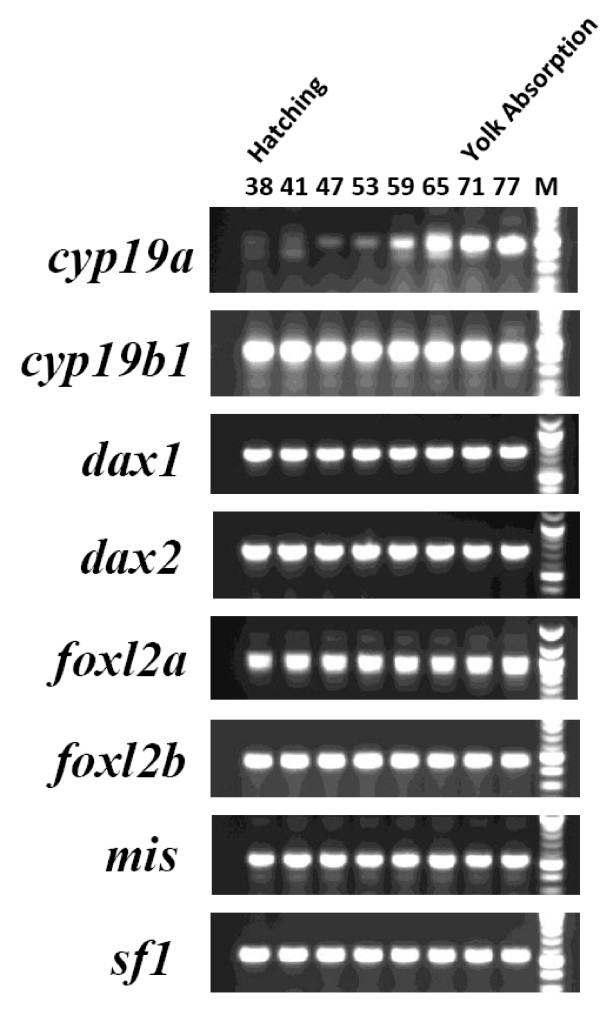
**Reverse-transcriptase PCR validation of gene-specific cDNA expression in larval and alevin stages of development**. Development stage indicated by day (Days 38 through 77) post-fertilization. For determining the lengths of each amplicon, the strongest marker bands indicate fragment lengths of 500 bp (*cyp19a*, *cyp19b1*, *foxl2b*, *mis *and *sf1*) or 1000 bp (*dax1*, *dax2 *and *foxl2a*).

Also of interest are the gene expression results for larvae and alevin between D38 to 77. It is known that *dax1*, *foxl2 *and *sf1 *expression correlates with transcription of *cyp19a *in many species [6,11,14,16,17,25,26]. We therefore were interested in examining the expression of these regulators in relationship to *cyp19a *transcription. Most if not all of the genes known to be involved in aromatase transactivation were expressed during this period (Figure 3). *cyp19b1 *was expressed weakly as early as D21 (Figure 2), but weak expression of *cyp19a *was not detected until at least D47 (Figure 3). The earliest "strong" detection of the expression of *cyp19a *was D59, nearly two months after the earliest expression of its regulators (Figure 3). Thus, although the genes (and presumably their protein products) important to *cyp19a *regulation were present, it was not until three weeks later (D59) that *cyp19a *transcription was strongly detected (Figure 3). This is similar to findings that were made for ovary-specific *cyp19a *expression in trout [5]. This time point may be considered a mark of the earliest molecular expression of steroidogenic and differentiation factors before morphologically distinguishable features of sex differentiation [5].

### Expression of sex differentiation factors in various adult tissues

Most of the information available on the genes we examined is from investigations done on the brain or gonadal tissue (eg; endocrine organs). We therefore explored the expression of each gene in twelve tissues extracted and prepared from three different male and female adult fish. Analysis of the RT-PCR results indicates that most of these genes were expressed quite broadly (see Additional file 2). We were most surprised to find the expression of *sf1*, *mis *and both *sox9 *genes across all tissues examined, particularly in the male. It was also interesting to compare the expression patterns between paralogs where it was possible to distinguish potential regulatory differences between *foxl2a *and *foxl2b*, and *cyp19a *and *cyp19b1 *transcripts (see Additional file 2). We also detected the expression of multiple products for *cyp19b1 *and *mis *and these amplicons were isolated, cloned and sequenced.

### Alternative splicing of mis transcripts

**Figure 4:**
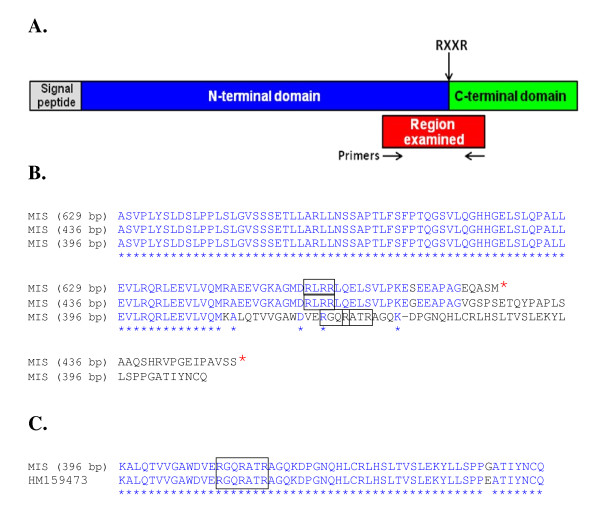
**Amino acid residue alignment of translated *mis *transcripts**. A. Schematic presentation of the organization of MIS and the region examined. The organization of MIS is based on alignments presented in Halm et. al. [27]. B. Partial amino acid residue alignment of translated MIS transcripts. Open reading frames based on sequence upstream from the C-terminal hormone portion of salmon MIS. Stop codons are denoted by red asterisks. Putative protease recognition motifs are boxed. C. Amino acid residue alignment of translated MIS (396 bp) and HM159473. The region bearing the protease recognition sites is aligned with the corresponding portion of HM159473 to highlight potential of *mis *(396 bp) to encode the C-terminal bioactive portion of salmon MIS.

MIS is a member of the TGF superfamily of proteins that share features that are conserved across many species from fish to mammals [27], each containing an N-terminal prodomain and a C-terminal domain that presents MIS. Proteolysis of the prodomain from the C-terminal domain at specific protease recognition motifs is required to release the hormone activity of MIS [27]. We isolated three transcripts of different sizes for *mis*. The primer set used was designed against sequence that spans a region of DNA that encodes potential enzymatic cleavage recognition residues and that is upstream from the bioactive hormone region of the molecule (nts 27979 to 28608 [GenBank:HM159473]) (Figure 4A). We found expression of a complete sequence (629 bp), and two alternatively-spliced transcripts of 436 and 396 bp in length (see Additional file 3). Similar alternatively-spliced variants of *mis *have also been characterized in the European sea bass [27]. The *mis *(629 bp) and *mis *(436 bp) transcripts terminate 22 and 47 codons downstream from their putative protease RLRR recognition motifs, respectively (Figure 4B). Translation of these transcripts thereby does not generate the C-terminal mature hormone portion of salmon MIS. Only *mis *(396 bp) is in-frame to encode the bioactive hormone portion of the gene (Figure 4C).

The features of the different *mis *transcripts may be important for different regulatory capacities, depending on the tissue in which they are expressed. For example, *mis *(629 bp) is expressed at relatively high levels in the muscle, skin, gill, spleen, brain and heart; *mis *(436 bp) is most "strongly" expressed in the gonad (as well as the kidney and eye), and *mis *(396 bp) is expressed at a relatively low level in most of the male tissues we examined (see Additional file 2). Other potential alternatively-spliced amplicons of *mis *are observed in several of the female tissues.

In the early development stages, expression of *mis *was first observed on D18 and we did detect the expression of multiple *mis *amplicons by D27 (data not shown). We attempted to clone and sequence each of these PCR products, but were successful in obtaining information for only the larger 629 bp transcript.

### Alternative splicing of cyp19b1 transcripts

During the process of preparing clones for sequencing to confirm their identities, we isolated and sequenced *cyp19b1 *transcripts of three different sizes from D38 larvae. Previous work demonstrated that at least five different *cyp19b1 *transcripts that bear differences in the lengths of their 5'-UTRs are generated in the brain and gonads of rainbow trout [23,28]. We now show similar results for the transcription of *cyp19b1 *transcripts with 5'-UTRs of a variety of lengths in Atlantic salmon.

**Figure 5:**
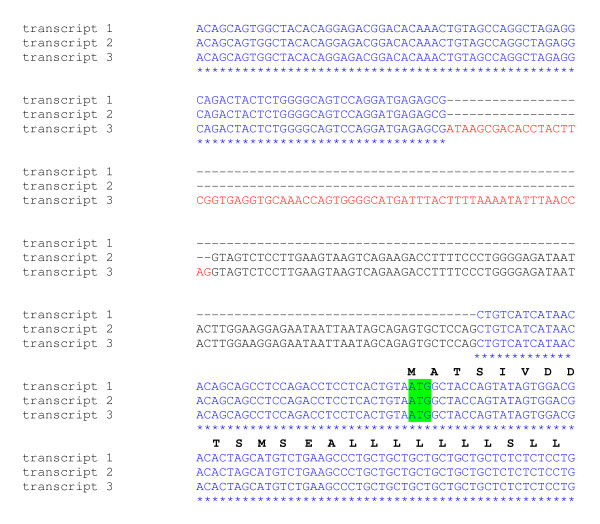
**Sequences of alternative 5'-ends found in three different salmon *cyp19b1***. transcripts. Transcripts 2 and 3 contain spliced sections of the 3'-end of intron 1 in comparison to transcript 1. The position of the initiator ATG start codon in exon 2 is highlighted.

**Figure 6:**
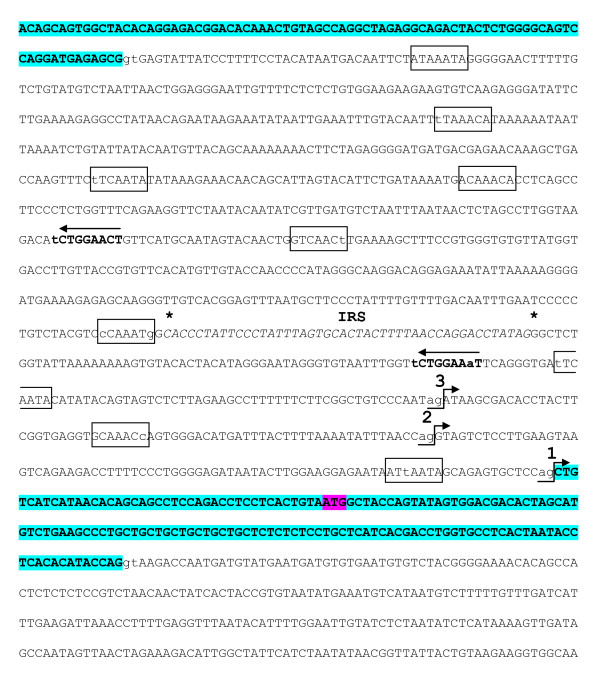
**Sequence of Atlantic salmon *cyp19b1 *exon 1, intron 1 and exon 2**. Boxed sequences represent putative binding elements of FOXL2. Arrows indicate the direction of potential regulatory motifs for SF-1 shown in bold. Numbers 1, 2 and 3 show the positions of each of the three AG acceptor 3'-splice sites within intron 1 that can be selected by the spliceosome to generate different 5'-UTRs in *cyp19b1*. An intronic repeat sequence (IRS) segment is shown in italics and enclosed by asterisks. Exons 1 and 2 are highlighted in bold and the ATG translation start site is shown in exon 2.

The salmon transcript 1 utilizes the more conventional intron 1/exon 2 splice sites and is devoid of intron 1 (Figure 5). Transcript 2 has been previously identified in the trout brain and gonads [23,28] and includes a portion of the 3'-end of intron 1 of the salmon *cyp19b1 *gene (Figure 5 and 6). Transcript 3 contains an extra 69 nts of intron 1, in addition to the most 3'-end of the intron that is included in the 5'-end of transcript 2. This is the first time that a *cyp19b1 *transcript using this more upstream splice site has been reported for a salmonid. Each of these distinct mRNAs use splice sites that follow the canonical pre-mRNA GT-AG rule for intron splicing [29]. It is interesting that the cryptic 3'-splice site utilized in transcript 2 is bypassed by the spliceosome in favour of the more upstream acceptor AG site in transcript 3. Of eight *cyp19b1 *clones isolated from amplifications of D38 cDNA, six were transcript 2 representatives. We presume that other examples of these partial intron 1-containing *cyp19b1 *transcripts exist. However, several of the larger (>521 bp), weaker *cyp19b1 *PCR products detected in the adult tissues were isolated, cloned and sequenced and determined to be non-specific amplifications (see Additional file 2).

Generally, strong 3'-splice sites in vertebrate intronic regions contain a pyrimidine-rich tract and a terminal AG [29,30]. These sequences are important for spliceosome recognition for precise intron removal [30]. Examining the 30 nts preceeding each of the three 3'-splice sites in intron 1 of *cyp19b1 *revealed that the conventional intron 1/exon 2 junction, the cryptic 3'-splice site and the most upstream splice site had 33% (1), 53% (2) and 63% (3) CT content, respectively (Figure 5). Interestingly, the splice site with the least pyrimidine content is that of the more conventional intron 1/exon 2 junction. It is therefore possible that the formation of this junction is the least favoured of the three splice sites presented here.

A comparison of the rainbow trout [GenBank:AJ716203] and Atlantic salmon *cyp19b1 *intron 1 shows strong conservation in the 5'-and 3'-ends (data not shown). The central region of the two introns contain indels and are less conserved. Interestingly, only one intronic repeat sequence (IRS) segment, previously reported as four contiguous blocks of 46 nts in the trout [23], was present in the salmon *cyp19b1 *intron 1 (Figure 6). The loss of the other three IRSs in the Atlantic salmon comprises almost half of the difference in the sizes between the introns (trout: 1264 bp; salmon: 964 bp).

The novelty of the cryptic 3'-splicing in the *cyp19b1 *intron 1 [23, 28 and refs therein] suggests this region is a target for regulators that bind signal elements within it to dictate distinct splicing events. Interestingly, a cluster of perfect and imperfect core recognition motifs for FOXL2 (5'-(G/A)(T/C)(C/A)AA(C/T)A-3' [16,25,26]) are located in the salmon *cyp19b1 *intron 1 (Figure 6). The IRS contains at least one potential half-site (AGGACCT) that could potentially bind estrogen receptor or retinoic acid receptor-retinoid X receptor heteromers. The IRS is also flanked by two everted SF-1 motifs that present only one mismatch to the consensus sequence YCAAGGYCR, where Y = T/C and R = G/A [31 and refs therein].

Recent viral and mammalian work has demonstrated that exonic splicing signals exist that can enhance (ESEs) or suppress (ESSs) spliceosome recognition for intron splice sites [30]. Although this class of RNA processing signal is ill-defined in fish, we previously identified several putative exonic splicing enhancers and suppressors in exon 2 of trout *cyp19b1 *[23]. These ESEs and ESSs are almost identical in the Atlantic salmon *cyp19b1 *exon 2 (data not shown), indicating that this exon contains significantly positioned splicing motifs that could also potentially direct the lengths of the 5'-end of *cyp19b1 *mRNAs.

### Conservation of nucleocytoplasmic transport signals in SOX9

**Figure 7:**
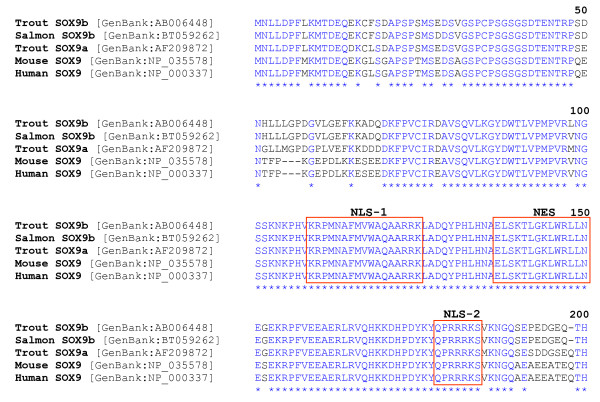
**Alignment of salmonid and mammalian SOX9 high mobility group (HMG) domains**. The mammalian and salmonid bipartite nuclear localization signal (NLS-1) and basic cluster NLS (NLS-2) are found in positions at the N- and C-termini of the HMG domain, respectively. The nuclear export signal (NES) is centrally located between the two NLSs. The C-terminal protein interface and transactivation domain residues are not shown.

The N-terminal high mobility group (HMG), DNA-binding domain of the characterized salmonid SOX-9 proteins are highly conserved, even in comparison to the mouse and human SOX-9 proteins (Figure 7). Signals important for the import and export of SOX-9 between the cytoplasm and the nucleus have been identified [32,33]. In particular, the bipartite and basic cluster nuclear localization signals (NLS-1 and -2) [32], as well as the leucine-rich nuclear export signal (NES) [33] are 100% identical across the species examined (Figure 7). The largest divergence between the mammalian and salmonid SOX-9 proteins occurs in the C-terminal non-HMG, protein-interface and transactivation domain (data not shown).

### BAC organization and regulatory regions

**Figure 8:**
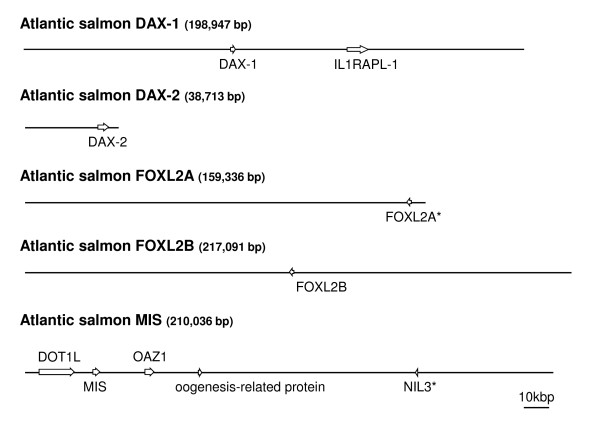
**The genomic organization of the genes that encode DAX-1, DAX-2, FOXL2A, FOXL2B and MIS**. Approximate sizes of the sequenced bacterial artificial chromosomes are indicated in parentheses. Each gene of interest and the genes that flank them are represented by boxes with transcriptional directions indicated by arrowheads. The introns for the genes are not shown. Pseudogenes are indicated by asterisks. IL1RAPL-1: X-linked interleukin-1 receptor accessory protein-like-1 precursor; DOT1L: DOT1-like histone H3 methyltransferase; NIL3: nuclear factor interleukin 3; OAZ1: ornithine decarboxylase antizyme-1.

In order to understand more fully the regulatory regions and chromosomal context of specific factors thought to be important in sexual differentiation processes in salmon, we isolated several BACs containing *dax1 *and *dax2*, *foxl2b *and *mis*. Once the BACs had been isolated and assembled, we examined approximately 3.5 kb of sequence in the proximal promoter regions and identified a number of different transcription factor response elements for each gene of interest (GOI). As well, the chromosomal environment in which each GOI resides is presented in Figure 8.

The mammalian *dax1 *promoter contains binding sites for OCT3/4, SOX-2, SF-1 and other regulatory factors [20]. We show both of the salmonid *dax *promoters contain binding elements for SF-1 and SOX-2 factors, but that only the *dax1 *proximal promoter contains OCT3/4 motifs (see Additional files 4 and 5). The possibility exists that these promoters could also be regulated by estrogen receptor since both have potential palindromic EREs. Other clear differences are discernable in the regulation of these two genes, most notably in the large regions of CT- and GA-rich regions in *dax2 *that are not present in *dax1 *(see Additional files 4 and 5). The GA-rich region is contiguous with a block of DNA that contains at least four perfect repeats, each 40 nts in length.

We could not identify any canonical SOX binding elements in the proximal *mis *promoter. However, we did detect two potential half-AREs and the presence of two SOX footprints [34] (see Additional file 6). Several OCT3/4, PPARE and WT-1 response elements were also identified. As well, typical GC boxes are located in the *mis *promoter (5'-GGGGCGGGGC-3') that could bind SP-1 and Kruppel-like factors [35].

We found a tetrapartite GCAT sequence in the *foxl2b *promoter that might bind SMAD factors. SMADs can act through GCNT, GCCG and GCAT sequence motifs [36]. Interestingly, the *foxl2b *promoter does not contain a strong consensus SF-1 binding motif. However, it is possible that a weaker site at -802 to -795 (5'-TCAGGCCA-3') could interact with SF-1 (see Additional file 7). We also provide evidence that FOXL2 may regulate its own expression through several binding sites present in its promoter. Numerous potential binding sites for ERE, RXR/RAR and WT-1 are also located in the *foxl2 *promoter.

**Figure 9:**
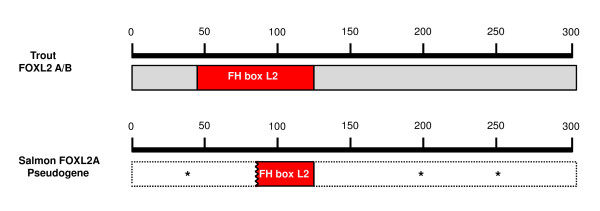
**A comparison of trout FOXL2A and B to salmon pseudogenic FOXL2 at level of translation**. An in-frame ATG start codon was not found for the salmon gene. Stop codons throughout the body of the putative transcript are denoted by asterisks. A small region of identity with the FH box domain is retained.

We also isolated a BAC containing the *foxl2a *pseudogene. The pseudogenic *foxl2a *mRNA, if transcribed, would share similarity with the characterized trout orthologs beginning in regions approximately 240 and 260 nts downstream from the start codon of the ortholog [GenBank:AY507927] and the diverged paralog [Genbank:AY507926] (see Additional file 8). We could find no inframe ATG initiation codons for the pseudogene in sequence upstream from these regions. Furthermore, multiple stop codons in the pseudogenic reading frame would disable any possible generation of a full-length protein. A comparison of the salmon pseudogene with the trout FOXL2 open reading frame shows a region of only short identity with the forkhead domain with the N-and C-terminal ends diverging completely (Figure 9). However, we did isolate an amplicon of 1010 nts that represents the salmon *foxl2a *based on sequence spanning from the 5'-UTR ending just short of the stop codon. We were unable to isolate the BAC bearing the viable *foxl2a *gene, but it has been mapped to linkage group (LG) 8 and *foxl2b *has been mapped to LG 5 in ASalBase [37]. The remaining BACs bearing the following genes have been localized to the following chromosomes by fluorescent *in situ *hybridization (FISH) techniques: *dax1*: chromosome 21 (LG14); *dax2*: chromosome 25 (LG20); *mis*: chromosome 10 (LG2) (see Additional files 9, 10 and 11).

### Transposon integration analysis

**Figure 10:**
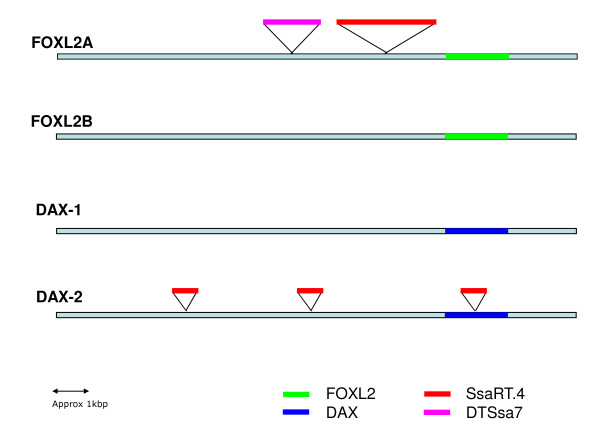
**Transposon integration in *dax *and *foxl2 *loci**. Insertion position of various transposon DNA sequences in *dax *and *foxl2 *loci are designated.

SsaRT.4, a non-LTR long interspersed nuclear element (LINE) sequence [38], is integrated approximately 1.3 kbp upstream from the transcription start site of the *foxl2a *pseudogene (Figure 10). This fragment is truncated at the 5'-end, consistent with partial retrotranscription. Additionally, we found a fragment of the Tc1-like transposon, DTSsa7 [38], inserted 800 bp upstream from the SsaRT.4 sequence. Interestingly, a SsaRT.4 sequence was also found inserted into the centre of the intron of *dax2 *(Figure 10). The GT-AG signals for intron splicing are approximately 600 and 1000 nts up- and downstream, respectively, from this integration. The *dax1 *paralog does not possess any introns. Truncated copies of SsaRT.4 are also present in the promoter of *dax2*. Both the *dax1 *and *foxl2b *promoters appear devoid of any transposon integrations.

## Discussion

### MIS

Postnatal expression of MIS in the early mammalian male gonad leads to male reproductive tract development mediated through regression of the female anlagen [39]. The importance of MIS to male sexual differentiation is demonstrated by the morphological and endocrine sex reversal of fetal ovaries exposed to MIS [40]. The expression of MIS has been associated with the downregulation of *cyp19a *in mammals [40], zebrafish [41] and sea bass [27].

We have observed expression of *mis *in immature and mature ovary and testis of both trout and salmon (data not shown). The activity of *mis *was absent only in precocious or prespawn trout testis. In this study, we show expression of *mis *during early development and in various adult salmon tissues (Figure 2 and 3; see Additional file 2). The expression of *mis *by D18 indicates the potential for a role early in salmonid development. Since MIS is a glycoprotein member of the TGF β family, it may serve in establishing and shaping different cellular phenotypes well before patterning of the reproductive tissues.

Since we observe expression of *mis *and the absence of *cyp19a *in most adult tissues (see Additional file 2), it could be suggested that MIS downregulates *cyp19a*. In contrast, the expression of *cyp19b1 *is evident in most adult tissues. Transactivation of *cyp19b1 *may not be reliant on gonadotropic inputs like *cyp19a *(ie; cAMP stimulation [17,23,40]), and therefore could be refractory to inhibition by MIS. Nevertheless, unlike in mammalian models where *mis *expression is absent during the onset of sex determination, *mis *expression is detected in the embryonic gonads of both trout [5,14] and tilapia [6]. Although the levels of expression in the female are much lower than in the male during the crucial sex determination window [5,6,14], it may still play a role in female development. For example, disruptions to MIS signaling have been shown to impact germ cell proliferation and cell-cycle timing in both sexes of medaka [42].

Furthermore, in mammalian models, *mis *is the downstream regulatory target of SOX-9 activity in the differentiating testis. However, expression of *sox9a *and *sox9b *occurs in both the trout male and female gonad during sex determination [5]. Part of the decoupling of MIS expression from SOX regulation, at least in salmonids, could be due to the lack of canonical SOX binding elements in the proximal *mis *promoter (see Additional file 6). Another control mechanism that may differ in fish that exists in mammals is that nuclear localization of SOX-9 occurs in the male (to promote subsequent MIS transcription), but is absent in the primordial female gonad [43]. The subcellular localization signals that have been shown to direct SOX-9 nucleocytoplasmic shuttling during mammalian male sexual differentiation [32,33] are highly conserved in salmonid SOX-9 proteins. Whether these signals are targets for sex-specific transport factors; and whether the *sox9 *paralogs are localized to different cell-types, and are involved in differentiation of Sertoli precursor cells in salmonids, still remains to be determined.

In mammals, the MIS precursor protein consists of two sections - the N-terminal prodomain and the C-terminal, mature hormone region [44]. The mammalian studies indicate that the MIS precursor protein is proteolytically cleaved at consensus RXXR sites [44]. It is not known if fish MIS proprotein is similarly proteolytically activated, but the organization and conservation of these motifs across many species does suggest similar mechanisms of hormone bioactivation [27]. Although the mammalian studies indicate that the N-terminal prodomain section of MIS serves only to stabilize and to target the processed hormone [44], our work suggests that some *mis *transcripts generate only truncated prodomain representatives. The function of these transcripts, in various tissues and during early development, remains to be determined. It is possible that the expression of transcripts that encode the MIS prodomain alone may add another level of control for tissue-specific regulation of MIS activity, or that they influence the bioactivities of other members of the TGF family, such as BMPs, activin or inhibin. Heterodimerization between various members of this large family of growth factors has been shown to direct different cell-patterning decisions [45 and refs therein, 46].

### DAX

DAX-1 has been described to be either a possible ovarian- or testicular-determining factor [12,14,44,47]. We previously found *dax1 *to be consistently expressed across all reproductive development stages examined in trout ovary and testis [23]. Although both the salmon *dax1 *and *dax2 *described here were expressed in all adult tissues (see Additional file 2), the appearance of *dax1 *was at least two weeks before *dax2 *in salmon embryos (Figure 2).

Although the interactions that promote the undifferentiated state in embryonic stem (ES) cells are well-established, the events that antagonize them to initiate cellular differentiation are poorly understood. The expression of *dax1 *has been associated with early mammalian ES cells in maintaining the undifferentiated state and to safeguard pluripotency [20,48]. OCT3/4 and SOX-2 are also considered "core" transcription factors in the maintenance of pluripotency [49]. It has been shown that these factors interact cooperatively to regulate *dax1 *expression in ES cells [20]. We found putative response elements for OCT3/4 and SOX factors in the *dax1 *and *dax2 *promoters (see Additional files 4 and 5), that implicate their role in the regulation of the salmonid genes as well.

It also has been proposed that SF-1 expression is not coupled to *dax1 *regulation in ES cells [20]. In this regard, it is also important to note the location of the SOX consensus binding elements in close proximity to the SF-1 motifs in both salmonid *dax *promoters (see Additional files 4 and 5). SOX binding of its response element may interfere with SF-1 binding during early development stages. We also show the expression of *sf1 *no earlier than D14, a period that succeeds a potentially important differentiation transition point (see below) (Figure 2).

Regulation of alternative splicing by DAX-1 and SOX factors may control stem cell differentiation, and initiation and development of the gonad [47]. It has been demonstrated that *dax1 *inhibits pre-mRNA splicing of target genes and that these activities can be restored by the addition of HMG-domain containing factors [47]. Moreover, it has been proposed that the control of splicing is exerted through competiton between DAX-1 and SOX proteins for splicing factor complexes [47]. Antagonism between DAX-1 (and OCT3/4 [20]) and SOX-2 (and other HMG-domain proteins?) [47] may be the mechanism whereby "stemness" versus differentiation-associated genes are regulated in ES cells. The decline (D10), absence (D12) and reappearance (D14) of *dax1 *we detected (Figure 2) may reflect a transition that results in differentiation of embryonic cell layers in specific developing organs; similar to the different cell lineages that have been shown to arise following *dax1 *downregulation in mouse [21,24].

### FOXL2

Both *foxl2 *transcripts were found to be expressed very early in development (Figure 2). In the male, we found *foxl2a *expression to be quite restricted in comparison to the *foxl2b *diverged paralog, which was present in most adult extragonadal tissues examined (see Additional file 2). In contrast, expression of *foxl2b *is quite restricted in the adult female tissues, essentially confined to the skin, gill and ovary. The expression of *foxl2b *in the testis was also very strong, correlating well with previous findings that showed *foxl2b *was present, with *foxl2a *absent, in later stages of testis development in trout [23]. Taken together, this may indicate that *foxl2b *is an important regulator of male-specific genes (eg; *cyp19b1*), at least later in development.

The expression of *foxl2a *is associated with upregulation of ovarian-specific *cyp19a *and with differentiation of the ovary in many species [6,14,16,17,25,26]. Upregulation of *foxl2 *genes have been correlated with *cyp19a *expression during ovarian differentiation in trout [14], Japanese flounder [17] and Nile tilapia [6,16]. Depending on the study, transactivation of *cyp19a *occurs directly through the FOXL2 binding elements or in concert with SF-1. SF-1-mediated regulation of aromatase activity is modulated by DAX-1, which binds SF-1 directly [11,15].

We previously implicated FOXL2 with the generation of *cyp19b1 *transcripts bearing 5'-ends of different lengths, potentially in complexes with SF-1 and DAX-1 [23]. At least two SF-1 binding sites and a number of potential FOXL2 response elements reside in the trout *cyp19b1 *intron 1. Because of well-characterized DAX-1 spliceosome activity (see above), and the association of both DAX-1 and SF-1 with FOXL2 at aromatase promoters in fish and mammals [6,11,15-17,25,26], we hypothesize that complexes of these gene products could also control splicing activity of *cyp19b1*.

This mechanism of regulatory control in the synthesis of these transcripts is further reinforced by our present characterization of the Atlantic salmon *cyp19b1 *intron 1 (Figure 6) which maintains many of the features described for the trout *cyp19b1 *intron 1 [23]. In accord with this model, we demonstrate the generation of differentially spliced, partial intron 1-containing *cyp19b1 *transcripts in salmon. We argue that if DAX-1, FOXL2 and SF-1 are known to interact at aromatase promoters, then nucleation at the *cyp19b1 *intron 1 is also plausible. Strong evidence exists that indicates that various transcription factors and nuclear hormone receptors can bind to RNA and regulate RNA splicing, migration and localization [50,51]. For example, both DAX-1 and SF-1 have been shown to bind steroid receptor activator [52], a RNA that acts as a bridge and platform to nucleate and stabilize transcription complexes. It has been proposed that nuclear receptors, such as SF-1, which can bind DNA as monomers, may recognize the YCAAGGYCR motif in RNA [52 and refs therein]. Among the various factors that bind RNA, recognition for YCAAGGYCR, as well as for other binding elements, may be through different mechanisms of interaction with the nucleic acid (e.g. zinc fingers, anti-parallel α-helices) [31,50,52].

### Aromatase

In this study, we confirmed the expression of alternatively-spliced *cyp19b1 *transcripts in only the larvae (D38) and gonad of salmon. However, expression of *cyp19b1 *transcripts of different sizes and abundances are evident in a broad range of adult tissues (see Additional file 2), such as muscle (521 bp), skin (452 bp) and spleen (367 bp) of the male. Whether differences in processing results in partitioning of specific *cyp19b1 *transcripts to distinct tissues remains to be explored.

We also show during early salmon development that at least a three-week delay in the transcription of *cyp19a *occurs in comparison to *cyp19b1 *(Figure 2 and 3). Part of these regulatory differences could be dictated by estrogenic or gonadotropic controls [23 and refs therein]. For example, unlike *cyp19b *promoters, *cyp19a *promoters lack consensus EREs for the fish species characterized to date [17,18,25,53-55]. Another important difference between these genes is that SOX binding elements, often many of them, are in close proximity to SF-1 binding motifs in teleost *cyp19a *promoters [18,25,53,54]. Competition between SOX factors and FOXL2 for access to their binding sites on *cyp19a *promoters could be key to ovarian-specific aromatase synthesis. This is compatible with recent work that shows that the mammalian ovarian phenotype is mediated by cooperative FOXL2 and ER repression of *sox9 *transcription [22]; interactions that are opposed by the binding of SRY/SF-1 and SOX-9 in the male pathway [19]. Further work is needed to define the precise roles that FOXL2A and FOXL2B play in activation of the two salmonid *cyp19 *genes early in development, as well as their function in the transcription of other promoters.

### Genome rearrangements

There is a growing body of evidence that demonstrates that past transposon activity has led to salmonid genome remodeling [38,56]. We show the integration of SsaRT.4 very close to the transcription start site of the pseudogenic *foxl2a *(Figure 10). Although speculative, integration of SsaRT.4 into the *foxl2a *regulatory region may have led to the reorganization of the 5'-end and subsequent accumulation of stop codons into the coding region of this gene. Integration of SsaRT.4 into the *dax2 *intron also provides an example of how transposon activity can rearrange genes; potentially leading to the creation of introns and indicating how genes can acquire new functions over time.

## Conclusion

We have found expression of many factors classically associated with sex differentiation in embryonic stages well before sex fate determination. Their expression early in development points to the potential for function during embryogenesis. Expression of these regulators in diverse adult tissues also indicates their potential role in transcription programs independent of steroidogenesis and gonadogenesis. Further investigation is required to determine if these genes are translated in the embryonic and adult stages of development and to localize their expression to specific cell-types. Whether these genes are associated with stem cells, or with other differentiation factors in networks that influence cell-patterning throughout development, remain to be unequivocally determined.

## Methods

### Animals and Sampling

Treatment of the fish used in this study was in compliance with the regulations of the University of Victoria Animal Care Committee. Eggs from Atlantic salmon (McConnell (Mowi)) were obtained in November, 2009 from Marine Harvest United Hatchery (Fanny Bay, B.C., Canada). The eggs were fertilized by gently mixing the eggs and milt by hand and then washed with partial exchanges of water. Approximately 2000 fertilized eggs were then transferred and placed in Heath trays (Marisource) at the University of Victoria. The embryos and larvae were raised in fresh water at a temperature of 12°C and a flow rate of 200 liters/h. Fry were transferred from the Heath trays to 30-liter holding tanks following yolk sac absorption.

The day of fertilization was Day (D) 0, and hatching and yolk sac absorption occurred between D38 to D40 and D68 to D70, respectively. Whole embryos (n = 20) were collected every other day for three weeks. Larvae were collected every third day and then alevin and fry every sixth day for the remainder of the study. Samples were directly placed into dry ice and stored at - 80°C until RNA extraction.

The number of mortalities were counted on each collection day. We also took several pictures using a Nikon D300 s with a 105 mm 2.8 G_ED lens to record the progress of the development of the fish.

Twelve different Atlantic salmon (Mowi stock) tissues from three individual adult male and female fish were provided by Fisheries and Oceans Canada (West Vancouver Laboratories, West Vancouver, British Columbia). The six fish were euthanized, followed by rapid dissection of tissues (kidney, muscle, skin, gut, gill, spleen, brain, heart, gonad, liver, eye and pyloric caeca). The tissues were flash frozen in liquid nitrogen or dry ice and stored at -80°C until RNA extraction.

### Reverse transcription and cDNA amplification

Total RNAs were extracted in TRIzol reagent (Invitrogen) by mixer-mill homogenization (Retsch) and spin-column purified using RNeasy Mini kits (Qiagen). The RNA from three whole embryos or two larvae or alevins was pooled for D2 to D20 and for D21 to D77 collections, respectively. RNA was extracted from portions of each adult tissue described above for each different fish. Each RNA sample was then quantified and quality-checked by spectrophotometer (NanoDrop Technologies) and agarose gel, respectively.

The cDNAs were synthesized in 25-μL reactions from 200 ng to 1.0 μg total RNA using oligo(dT)_15 _(Promega) and Supercript II RNase H^- ^reverse transcriptase according to the manufacturer's instructions (Invitrogen). The reactions were incubated at 37°C for 90 min and the transcriptase heat-inactivated at 70°C for 30 min. Approximately 100 to 200 ng of cDNA was used in each 25-μL PCR reaction containing 1.25 U Taq polymerase, 1 × Taq buffer, 1.25 mM MgCl_2_, 10 mM dNTPs (Invitrogen) and 15 pmol of each gene-specific 5'- and 3'-primer. Each PCR was carried out under the following cycling parameters: 94°C for 2 min, then 35 cycles of 94°C for 30 sec, 55°C for 30 sec (or 58°C for *sox9b*), and 72°C for 1 min using a Perkin Elmer GeneAmp 9600. The PCR products were separated by electrophoresis on 1.0 to 1.5% agarose gels and photographs were stored using an UVP GelDoc-It documentation system (Ultraviolet Products).

Genomic DNA to enable isolation of the *cyp19b1 *intron 1 was extracted from Atlantic salmon spleen using a DNeasy Blood and Tissue kit following instructions of the manufacturer (Qiagen).

### PCR analysis of sex differentiation factor expression in adult tissue

PCRs were performed with an initial denaturation step of 2 min at 95°C and then 35 cycles as follows: 30 sec of denaturation at 95°C, 30 sec of annealing at 55°C and 1 min of extension at 72°C using a Peltier Thermal Cycler PTC-225 (MJ Research). Amplicons for each gene of interest (GOI) were visualized on 1.0% agarose gels stained with ethidium bromide and images were stored using an UVP GelDoc-It documentation system (Ultraviolet Products). The intensity of the amplification products were semiquantitatively measured using ImageJ software [57] and divided by the intensity of the respective ubiquitin signals. The data generated was graphed in Microsoft Excel 2007 and expressed as the mean +/-SE of three individuals studied.

### Gene identification and primer design

The primers used to amplify trout ovarian aromatase (*cyp19a*), brain aromatase (*cyp19b1*), dosage-sensitive sex reversal, adrenal hypoplasia congenital, critical region on the X-chromosome, gene-1 and -2 (*dax1 *and *2*), forkhead box L2 ortholog (*foxl2a*), forkhead box L2 diverged paralog (*foxl2b*), Mullerian inhibiting substance (*mis*), steroidogenic factor-1 (*sf1*), sex-determining region of the Y chromosome (SRY)-related high mobility group (HMG) box (SRY box) Sox9a (*sox9a*), Sox9b (*sox9b*), and ubiquitin (*ubiq*) were designed specifically against the sequences provided for each gene obtained from http://www.ncbi.nlm.nih.gov. GenBank accession numbers, primer sets and product sizes for each GOI are shown in Additional file 12. The primer sets for each target gene were designed using Primer3 software [58] and purchased from Integrated DNA Technologies. The specificity of each primer set and the identity of each amplicon were confirmed by subsequent cloning into pCR2.1-TOPO vector (Invitrogen) and sequencing.

### BAC screening and sequencing

Atlantic salmon (AS) CHORI-214 [59] bacterial artificial chromosome (BAC) libraries were obtained from BACPAC Resources, Children's Hospital Oakland Research Institute (CHORI) [60]. AS BAC library filters were hybridized with 75-mer oligonucleotide probes for each GOI (Integrated DNA Technologies) that were 5'-end-labeled with ^32^P-ATP using T4 polynucleotide kinase (Invitrogen). Filter hybridizations were conducted as described by CHORI [60].

Confirmation of each GOI-containing BAC was performed by comparisons of *Hind*III restriction digests of the isolated clones to *in silico *digests for each BAC. BAC clones were chosen based on the physical BAC fingerprint map for Atlantic salmon that is publicly available on the internet Contig Explorer (iCE) version 3.5 [61]. The BAC end sequence information, that is available in ASalBase [37], was also used for selection of the BAC clones.

### BAC preparation and library construction

BAC DNA was isolated by an alkaline lysis procedure using Large Construct Isolation Kit (Qiagen) following the manufacturer's protocol. The isolated BAC DNA was nebulized and the DNA ends were made blunt by filling with T4 polymerase. The blunt-ended, repaired DNA was size fractioned by electrophoresis and the gel region corresponding to 1.5 to 3.0 kb was excised and gel purified (Qiagen). The fragments were blunt-end ligated into *Sma1*-cut M13mp19 vector and transformed into electrocompetent DH5α *E. coli *cells using a Bio-Rad Gene Pulser system. Library quality was evaluated and high redundancy plating was followed by large-scale colony picking (Genetix). Extracted recombinant plasmid templates were sequenced on an ABI 3730 DNA sequencer.

### BAC alignment and annotation

Bases were called using PHRED [62,63]. High quality sequence reads were assembled using PHRAP [64] and then viewed and edited using Consed [65]. Some gaps in BAC assembly were filled by designing primers to the contiguous sequence ends, followed by amplification of the BAC region by PCR and subsequent cloning and sequencing of the fragments.

Each BAC has been deposited in GenBank as follows: BAC 33H18 containing *dax1*

[GenBank:HM159469]; BAC 105M10 containing *dax2 *[GenBank:HM159470]; BAC 217E24 containing *foxl2a *ortholog (pseudogene) [GenBank:HM159471]; BAC 261D01 containing *foxl2b *diverged paralog [GenBank:HM159472], and BAC 19C14 containing *mis *[GenBank:HM159473].

BAC clone sequence data were annotated using BLAST homolog searches and EMBOSS programs package [66]. Alignment of cDNA sequences against genomic sequences was done by Sequin [67], which was obtained from NCBI. Identification of transposon insertions was performed using Dotter plots [68], comparing each GOI-containing BAC with salmon transposon sequences [38].

### Fluorescent in situ hybridizations

BAC DNA was labeled with Spectrum Orange using a nick translation kit (Abbott Molecular). Human placental DNA (2 μg) and Cot-1 DNA (1 μg, prepared from Atlantic salmon) or Cot-1 DNA (1 μg, prepared from rainbow trout) were added to the probe mixture for blocking. Hybridizations were carried out at 37°C overnight and post-hybridization washes were as recommended by the manufacturer (Abbott Molecular) with minor modifications [69]. Antibodies to Spectrum Orange (Molecular Probes) were used to amplify the signal. Slides were counter-stained with 4',6-diamidino-2-phenylindole (DAPI) at a concentration of 125 ng DAPI in 1.0 mL antifade solution. Images were captured with a Jai camera and analyzed with Cytovision Genus (Applied Imaging, Inc.) software. Chromosomes were arranged according to size within the metacentric/submetacentric and acrocentric groups [70].

The chromosome preparations were obtained from blood of the Norwegian strain of Atlantic salmon (2N = 58) by methods described previously for salmonid fishes [70]. Briefly, the buffy coat was isolated from whole blood and placed in MEM media with pen-strep, L-glutamine, 10% fetal calf serum and 200 µg/ml LPS and cultured for 6 days at 20°C. Cells were collected by centrifugation and re-suspended in 0.075 M KCl for 30 min, then fixed in 3:1 methanol acetic acid. Cell suspensions were dropped onto clean slides and allowed to dry on a slide warmer with humidity at 40°C.

### Promoter analysis

To identify the location of potential transcription factor binding elements in the promoters of our GOI, we preliminarily used MatInspector (Genomatix Software GmbH). Our designation of OCT3/4 consensus binding motifs 5'-ACACGCAT-3' or 5'-ATTTGCAT-3' are based on work done in ES cells [20,71]. The HMG domain that binds to DNA is highly conserved among all SOX factors (5'-(A/T)(A/T)CAA(A/T)G-3') [72]. The SOX footprints that we also include in our analysis are based on work done on mammalian *mis *and *cyp19 *promoters [34]. We use the term "immediate early response" element (IER) to identify response elements (5'-GCG(G/T)GGGCG-3') that potentially could bind early response products of genes such as EGR-1 and WT-1 [73]. Sequences presumed or demonstrated to bind CRE, ERE, FOXL2, PPARE, RXR/RAR and SF-1 in other fish species are also presented for each promoter examined [16-18,25,53-55,74].

## Abbreviations

**ARE**: androgen response element; **BMP**: bone morphogenetic protein; **CRE**: cyclic AMP response element; **EGR-1**: early growth response-1; **ERE**: estrogen response element; **OCT3/4**: octamer binding factor 3/4; **PPARE**: peroxisome proliferator-activated receptor element; **RXR/RAR**: retinoid X receptor/retinoic acid receptor; **SMAD**: mothers against decapentaplegic; **SP-1**: stimulatory protein-1; **TGFβ**: transforming growth factor β; **WNT**: wingless-related MMTV integration site.

## Authors' contributions

KRVS designed and coordinated the study, performed GOI-specific PCRs of early development stage cDNAs, promoter analysis and drafted the manuscript. MY performed GOI-specific PCRs of adult tissue cDNAs and semi-quantitative analysis. RY performed BAC sequence data analysis and annotations. JdB performed transposable element analysis. LR performed hybridizations to isolate and identify GOI-containing BACs. SS, AR and EBR performed BAC clone preparation and sequencing. RBP performed FISH on BAC DNA. WSD and BFK obtained funding and contributed to experimental design and analysis. All authors read and approved the final manuscript.

## Supplementary Material

Additional file 1 **Graphical presentation of number of mortalities found throughout early development**. Day represents post-fertilization age. Click here for file

Additional file 2 **cDNA expression profiles for ten genes of interest in twelve different adult tissues**. cDNA expression profiles for ten genes of interest (GOI) in twelve different tissues: 1: kidney, 2: muscle, 3: skin, 4: gut, 5: gill, 6: spleen, 7: brain, 8: heart, 9: testis, 10: liver, 11: eye and 12: pyloric caecum. Semi-quantitative levels of expression were calculated for each GOI as signal intensity relative to ubiquitin levels for three individual fish. Mean values (bars) of three individuals plus standard error are shown. Click here for file

Additional file 3 **Alignment of partial *mis *transcripts**. Sequence alignment of *mis *cDNAs and amino acid residues based on sequence upstream from the C-terminal hormone portion of salmon MIS. A. The stop codon for *mis *(629 bp) is highlighted in red. The open reading frame of *mis *(396 bp) cDNA is in-frame for encoding the bioactive hormone portion of MIS and does not contain a stop codon. Two potential protease recognition motifs (RGQR AND RATR) for translated MIS (396 bp) are boxed. B. The stop codons for *mis *(629 bp) and *mis *(436 bp) are highlighted in red. Putative cleavage RLRR recognition sites are boxed. Click here for file

Additional file 4 **Proximal promoter sequences of the Atlantic salmon *dax1 *gene**. The potential binding elements of various transcription factors, TATA boxes, exon 1 and initiator methionine codon are labeled. Click here for file

Additional file 5 **Proximal promoter sequences of the Atlantic salmon *dax2 *gene**. The potential binding elements of various transcription factors, TATA boxes, exon 1 and initiator methionine codon are labeled. Click here for file

Additional file 6 **Proximal promoter sequences of the Atlantic salmon *mis *gene**. The potential binding elements of various transcription factors, TATA boxes, exon 1 and initiator methionine codon are labeled. Click here for file

Additional file 7 **Proximal promoter sequences of the Atlantic salmon *foxl2b *gene**. The potential binding elements of various transcription factors, TATA boxes, exon 1 and initiator methionine codon are labeled. Click here for file

Additional file 8 **Proximal promoter sequences of the Atlantic salmon pseudogenic *foxl2a *gene**. Potential TATA boxes and the 5'-end portion of a region of sequence similarity shared with rainbow trout *foxl2 *genes are labeled. Click here for file

Additional file 9 **Fluorescent *in situ *hybridization of *dax1*-containing BAC**. Click here for file

Additional file 10 **Fluorescent *in situ *hybridization of *dax2*-containing BAC**. Click here for file

Additional file 11 **Fluorescent *in situ *hybridization of *mis*-containing BAC**. Click here for file

Additional file 12 **Primer oligonucleotide sequences used in RT-PCRs**. Click here for file
