# Supplementary material for: Regulation and expression of sexual differentiation factors in embryonic and extragonadal tissues of Atlantic salmon
Source: BMC Genomics. 2011 Jan 13;12:31. doi: 10.1186/1471-2164-12-31 (PMC3034696; doi:10.1186/1471-2164-12-31)
Supplement: Additional file 2 — cDNA expression profiles for ten genes of interest in twelve different adult tissues. cDNA expression profiles for ten genes of interest (GOI) in twelve different tissues: 1: kidney, 2: muscle, 3: skin, 4: gut, 5: gill, 6: spleen, 7: brain, 8: heart, 9: testis, 10: liver, 11: eye and 12: pyloric caecum. Semi-quantitative levels of expression were calculated for each GOI as signal intensity relative to ubiquitin levels for three individual fish. Mean values (bars) of three individuals plus standard error are shown. [file 1471-2164-12-31-S2.PPT]

## Slide 1
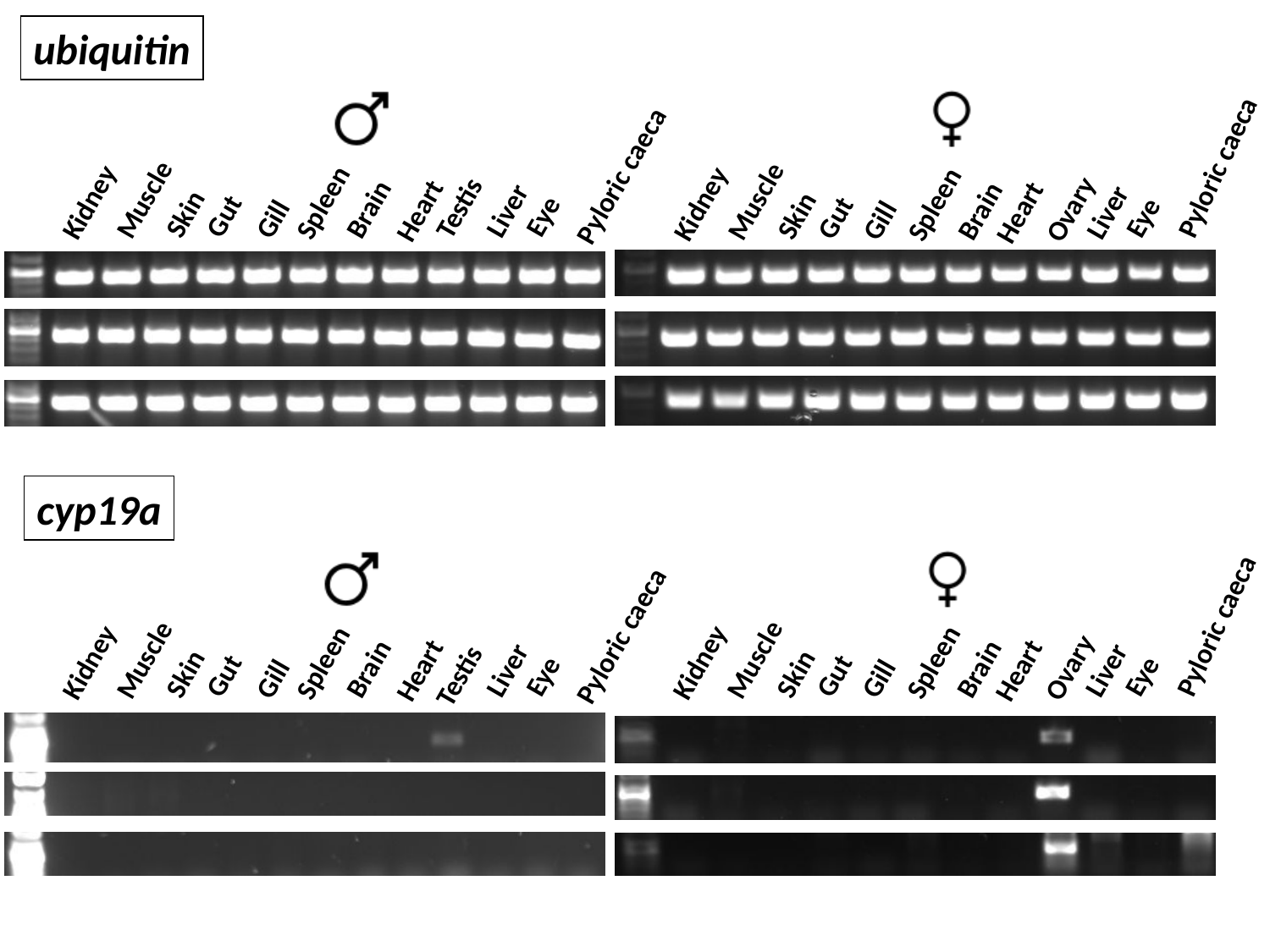

ubiquitin
Pyloric caeca
Muscle
Kidney
Spleen
Ovary
Brain
Liver
Heart
Skin
Gut
Eye
Gill
Pyloric caeca
Muscle
Kidney
Spleen
Testis
Brain
Liver
Heart
Skin
Gut
Eye
Gill
cyp19a
Pyloric caeca
Muscle
Kidney
Spleen
Ovary
Brain
Liver
Heart
Skin
Gut
Eye
Gill
Pyloric caeca
Muscle
Kidney
Spleen
Brain
Liver
Heart
Skin
Testis
Gut
Eye
Gill

## Slide 2
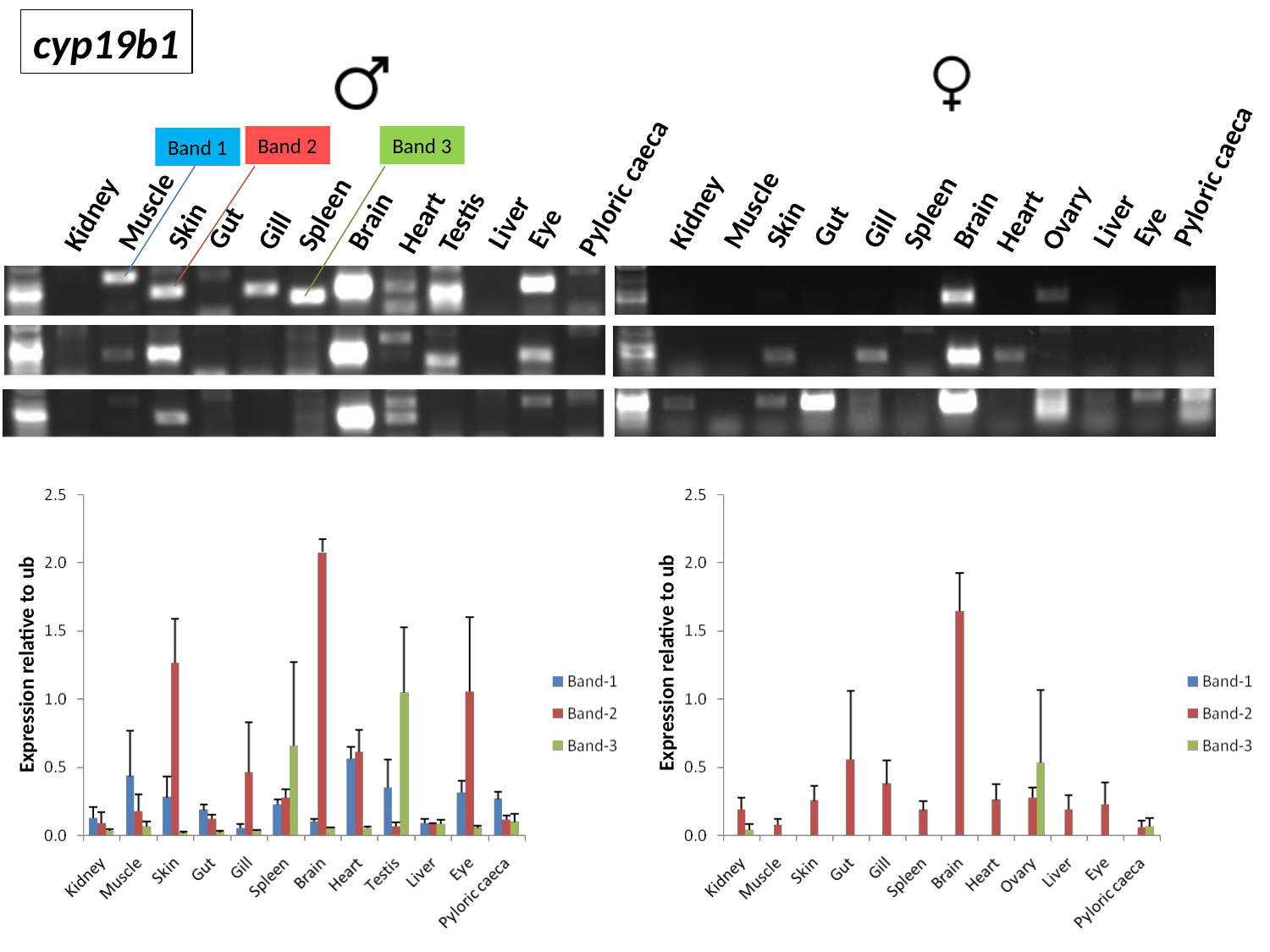

cyp19b1
Pyloric caeca
Muscle
Kidney
Spleen
Ovary
Brain
Liver
Heart
Skin
Gut
Eye
Gill
Band 2
Band 3
Band 1
Pyloric caeca
Muscle
Kidney
Spleen
Brain
Testis
Liver
Heart
Skin
Gut
Eye
Gill
Expression relative to ub
Expression relative to ub

## Slide 3
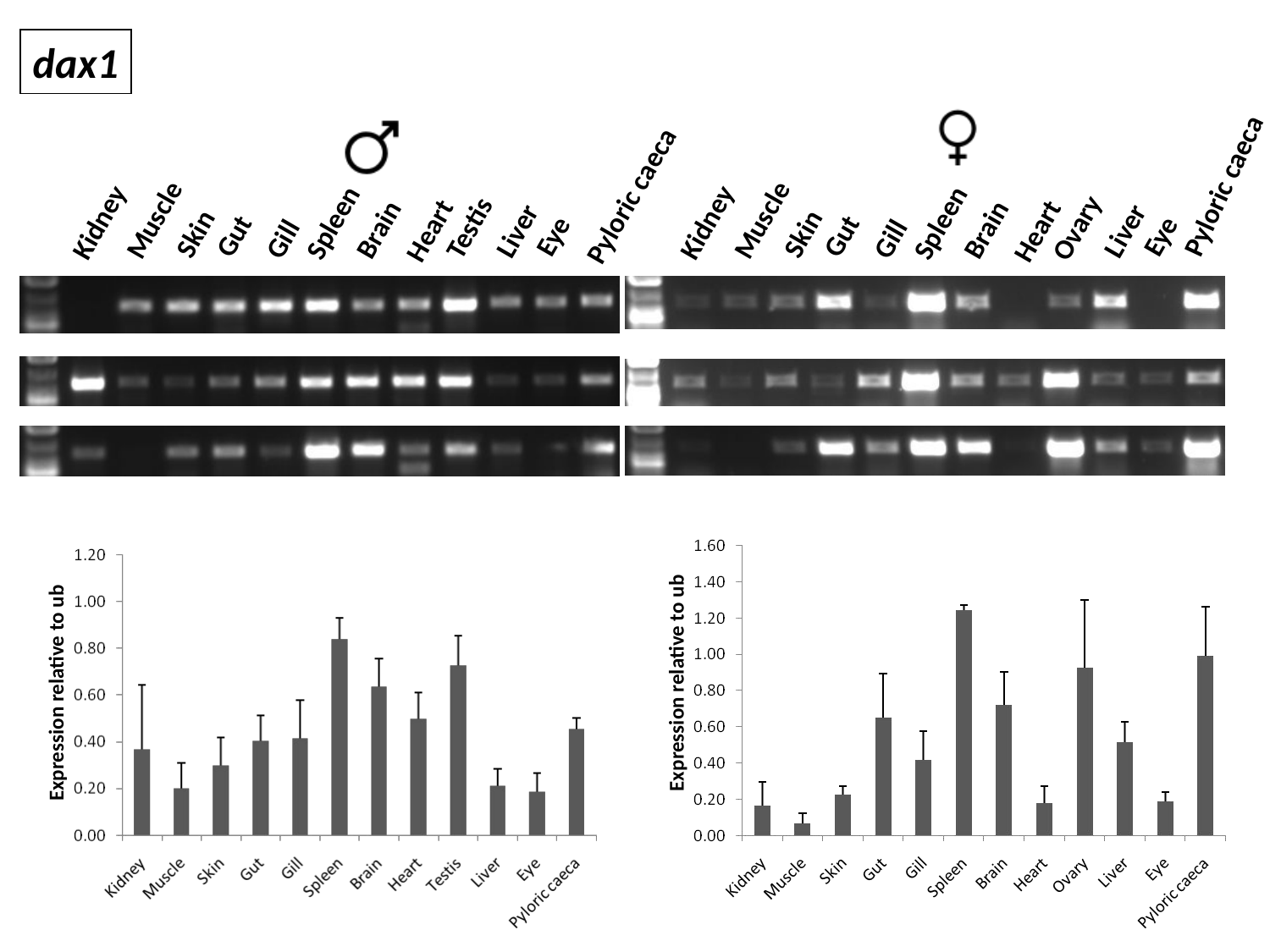

dax1
Pyloric caeca
Muscle
Kidney
Spleen
Ovary
Brain
Liver
Heart
Skin
Gut
Eye
Gill
Pyloric caeca
Muscle
Kidney
Spleen
Testis
Brain
Liver
Heart
Skin
Gut
Eye
Gill
Expression relative to ub
Expression relative to ub

## Slide 4
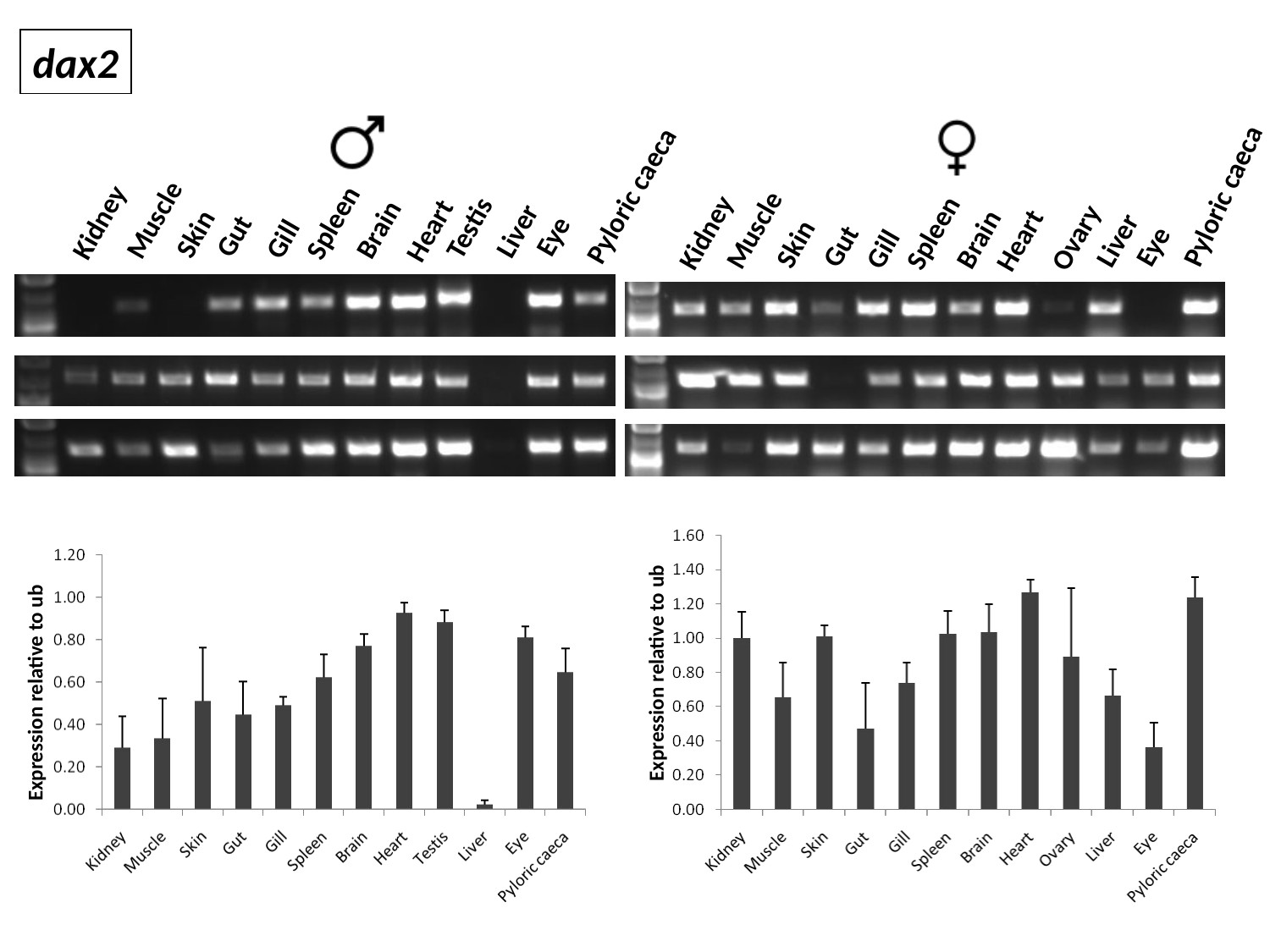

dax2
Pyloric caeca
Muscle
Kidney
Spleen
Ovary
Brain
Liver
Heart
Skin
Gut
Eye
Gill
Pyloric caeca
Muscle
Kidney
Spleen
Testis
Brain
Liver
Heart
Skin
Gut
Eye
Gill
Expression relative to ub
Expression relative to ub

## Slide 5
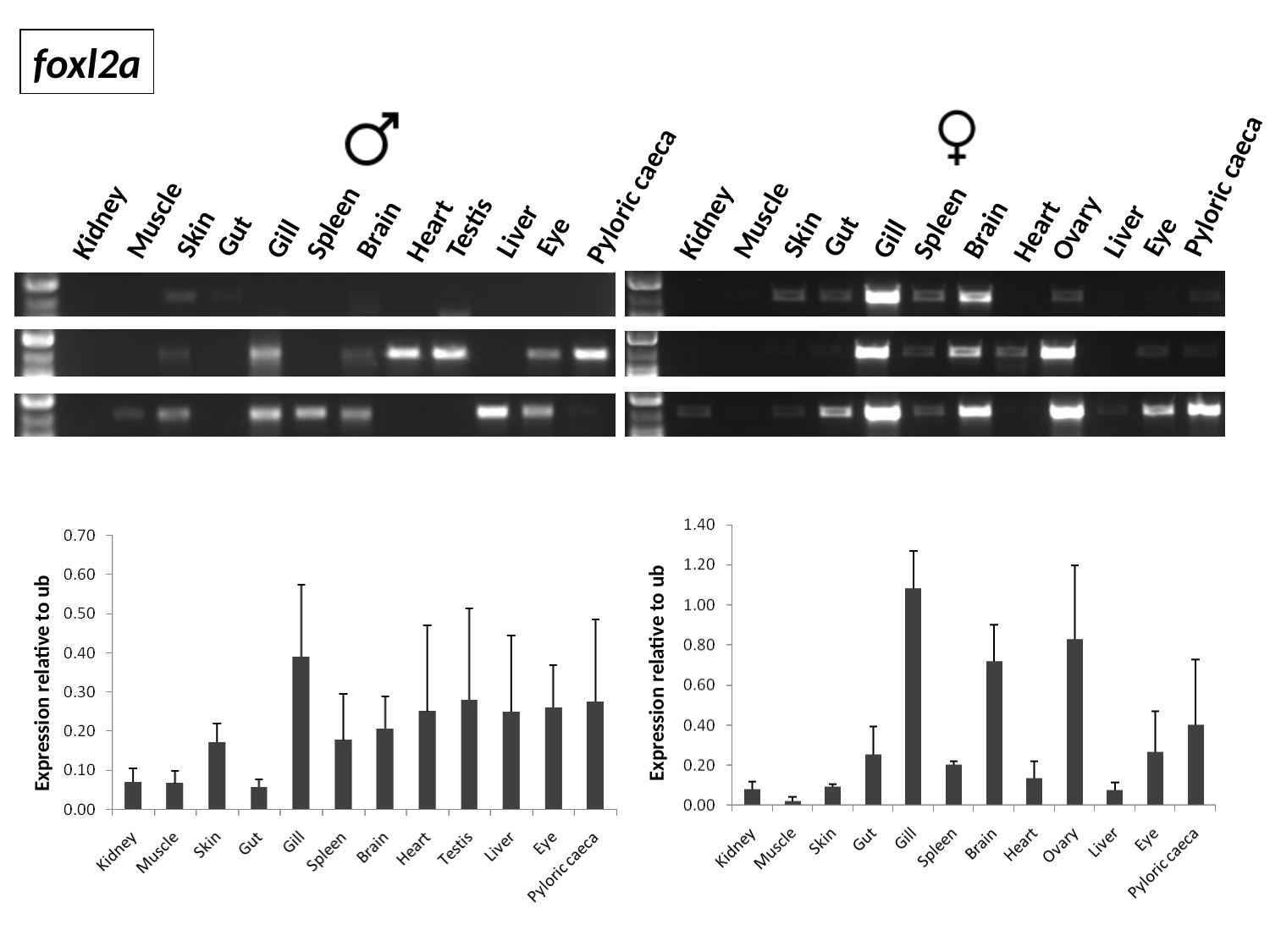

foxl2a
Pyloric caeca
Muscle
Kidney
Spleen
Ovary
Brain
Liver
Heart
Skin
Gut
Eye
Gill
Pyloric caeca
Muscle
Kidney
Spleen
Testis
Brain
Liver
Heart
Skin
Gut
Eye
Gill
Expression relative to ub
Expression relative to ub

## Slide 6
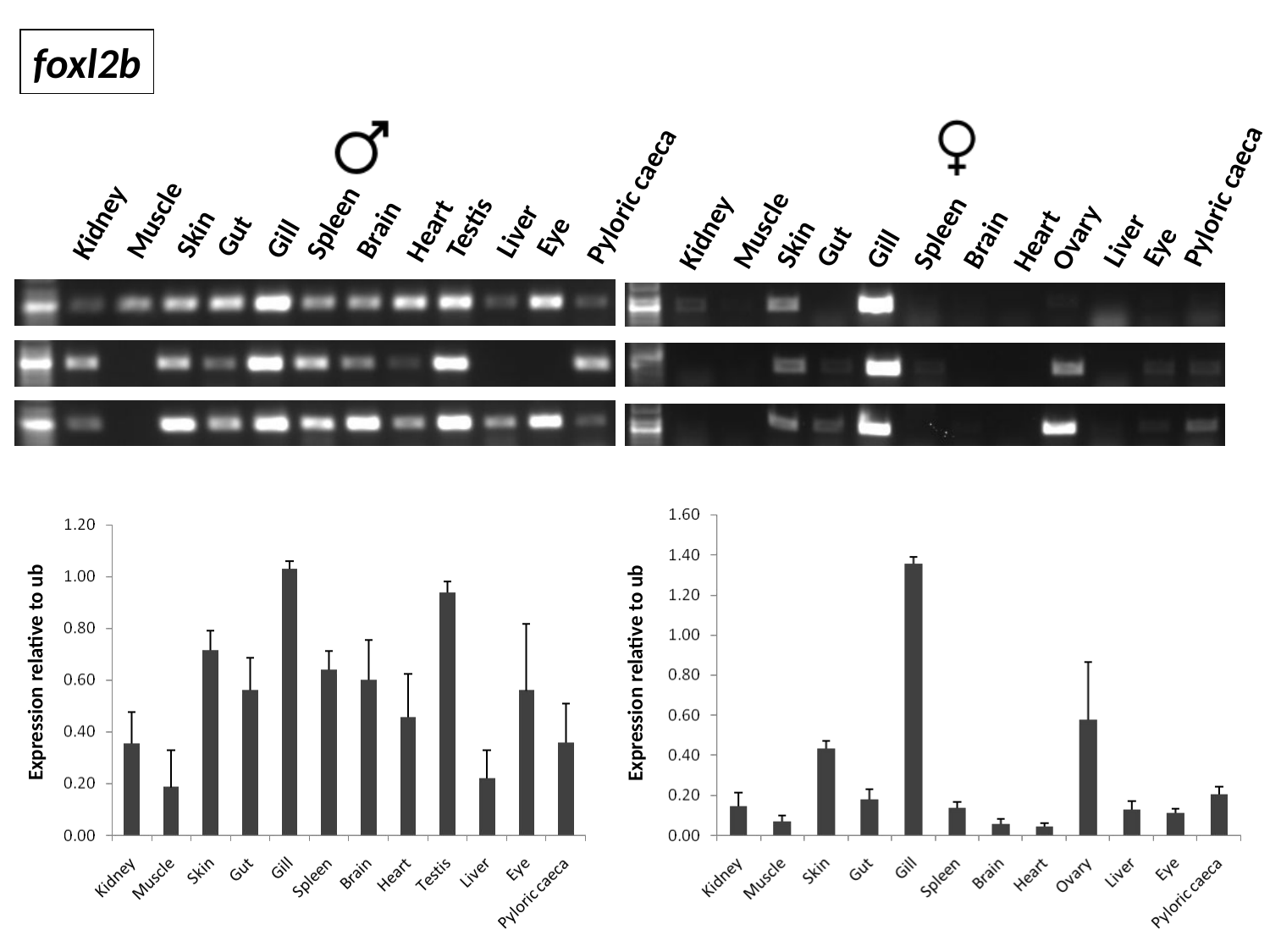

foxl2b
Pyloric caeca
Muscle
Kidney
Spleen
Ovary
Brain
Liver
Heart
Skin
Gut
Eye
Gill
Pyloric caeca
Muscle
Kidney
Spleen
Testis
Brain
Liver
Heart
Skin
Gut
Eye
Gill
Expression relative to ub
Expression relative to ub

## Slide 7
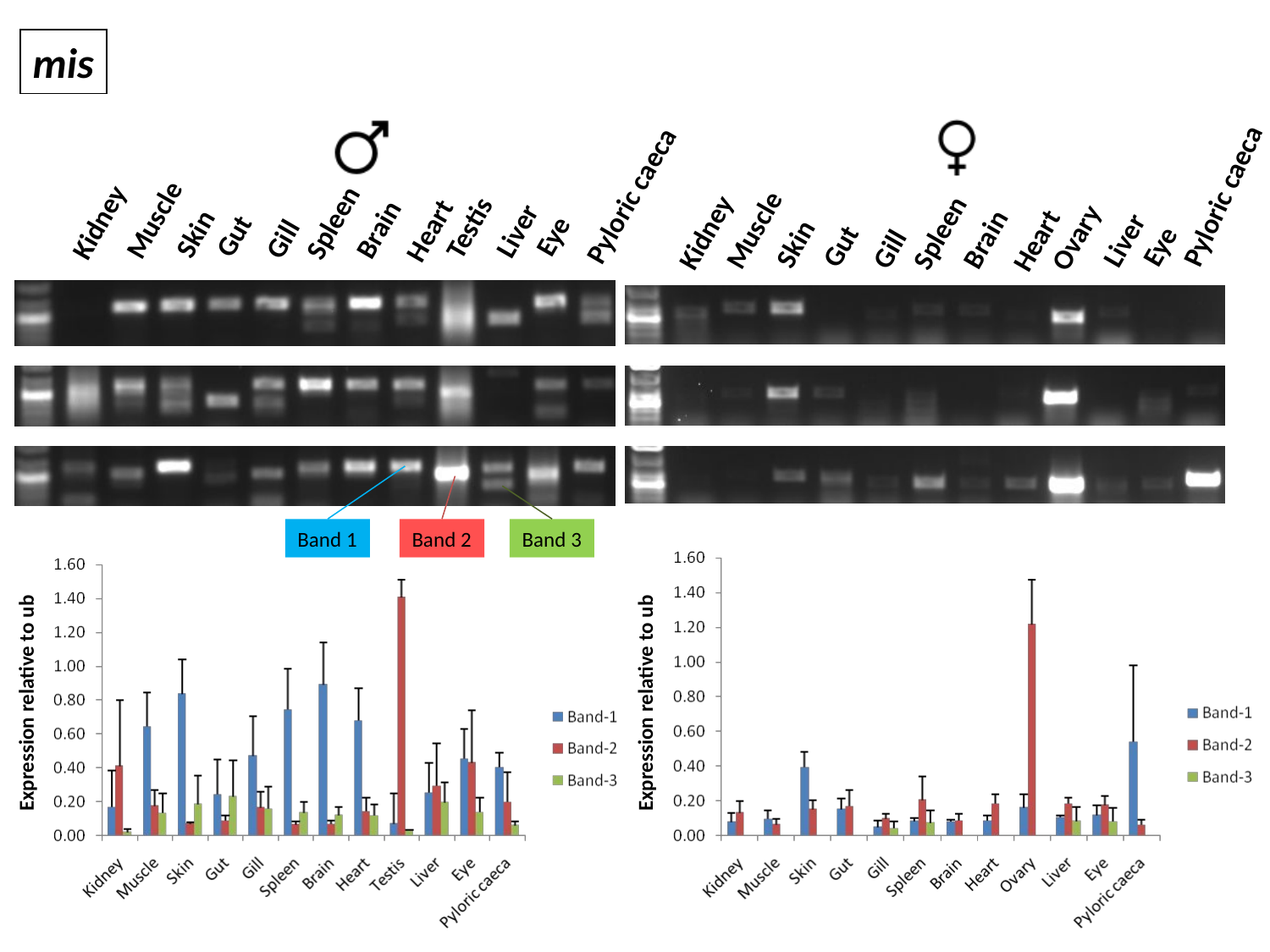

mis
Pyloric caeca
Muscle
Kidney
Spleen
Ovary
Brain
Liver
Heart
Skin
Gut
Eye
Gill
Pyloric caeca
Muscle
Kidney
Spleen
Testis
Brain
Liver
Heart
Skin
Gut
Eye
Gill
Band 1
Band 2
Band 3
Expression relative to ub
Expression relative to ub

## Slide 8
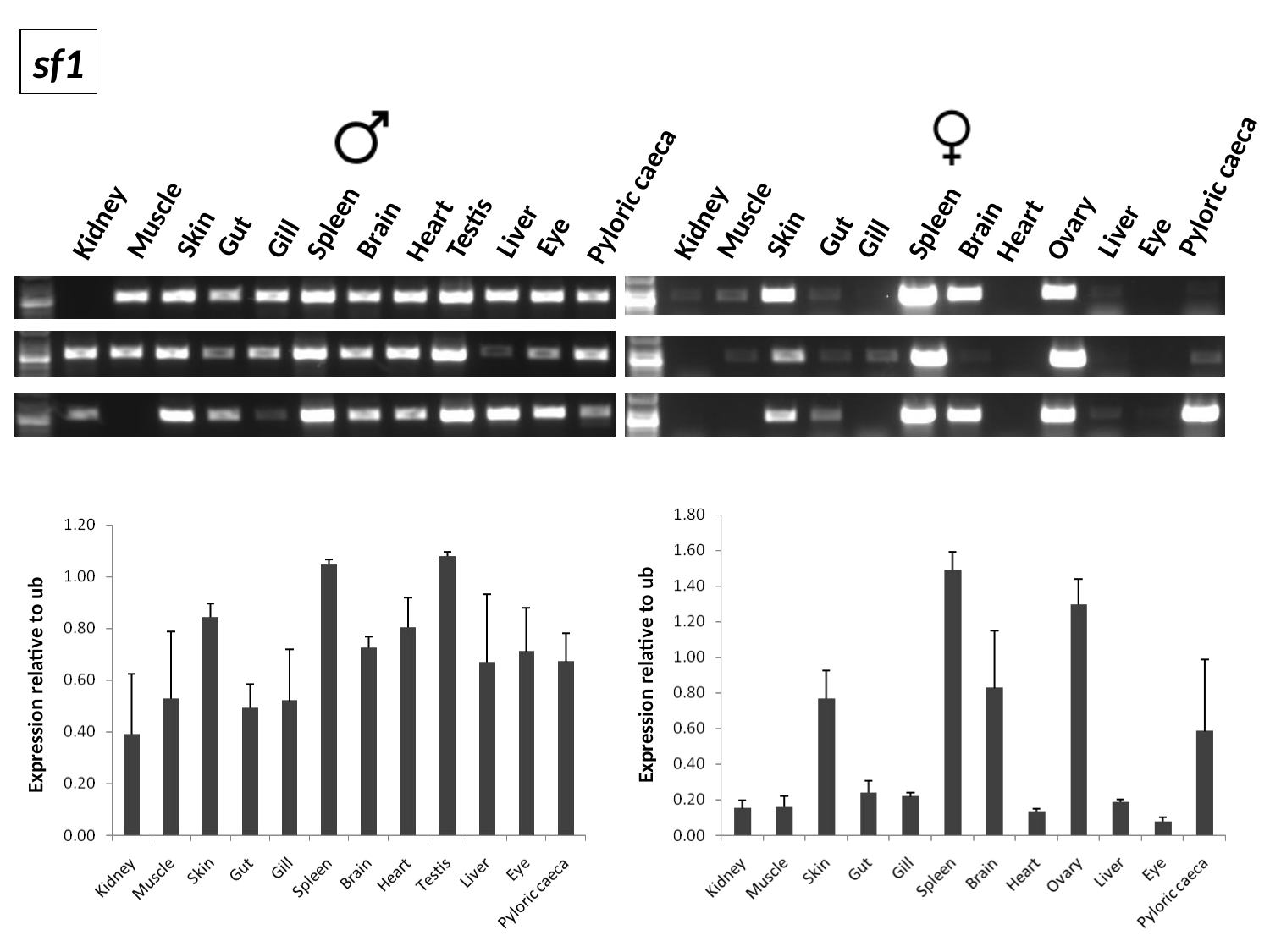

sf1
Pyloric caeca
Muscle
Kidney
Spleen
Ovary
Brain
Liver
Heart
Skin
Gut
Eye
Gill
Pyloric caeca
Muscle
Kidney
Spleen
Testis
Brain
Liver
Heart
Skin
Gut
Eye
Gill
Expression relative to ub
Expression relative to ub

## Slide 9
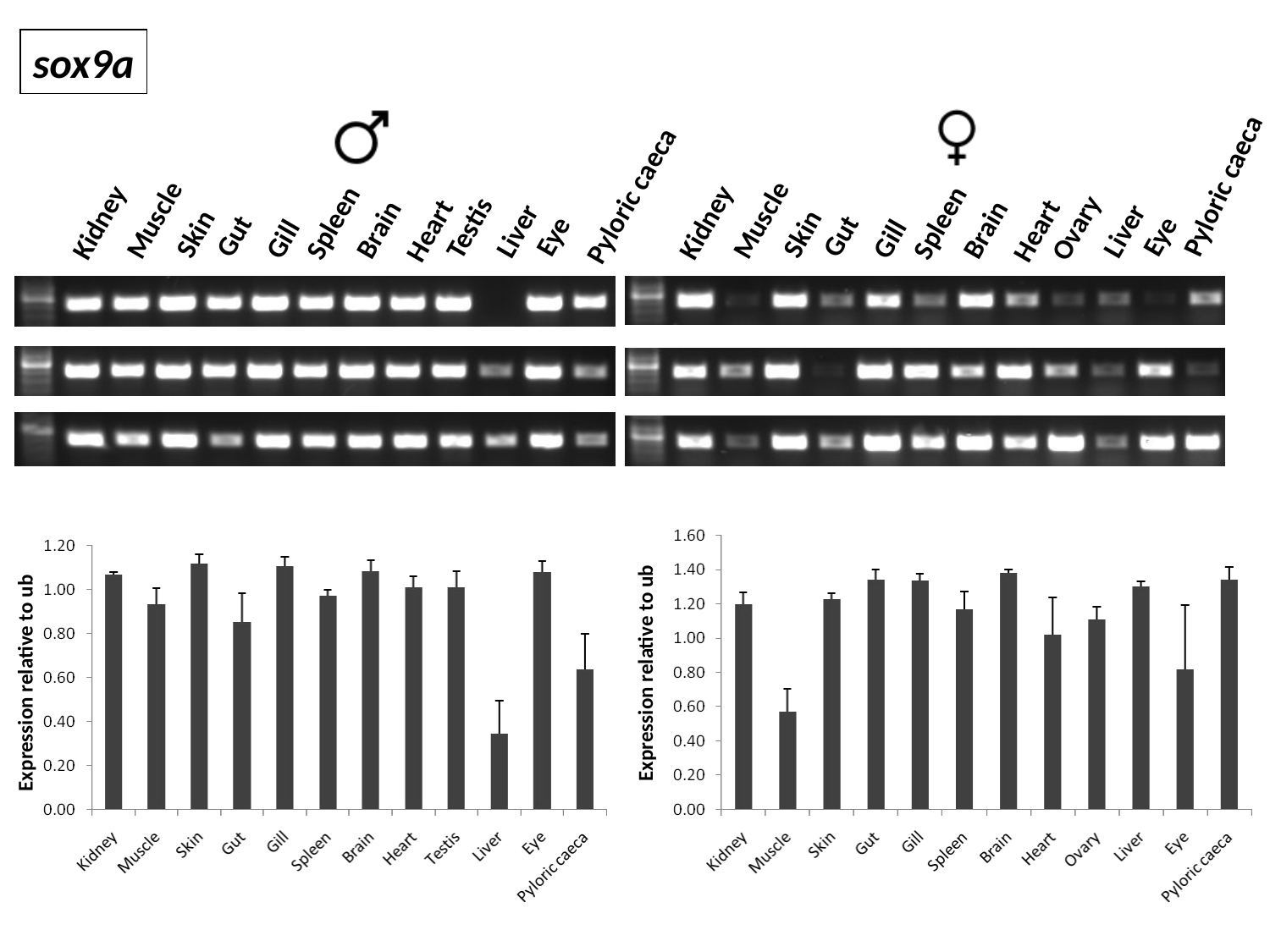

sox9a
Pyloric caeca
Muscle
Kidney
Spleen
Ovary
Brain
Liver
Heart
Skin
Gut
Eye
Gill
Pyloric caeca
Muscle
Kidney
Spleen
Testis
Brain
Liver
Heart
Skin
Gut
Eye
Gill
Expression relative to ub
Expression relative to ub

## Slide 10
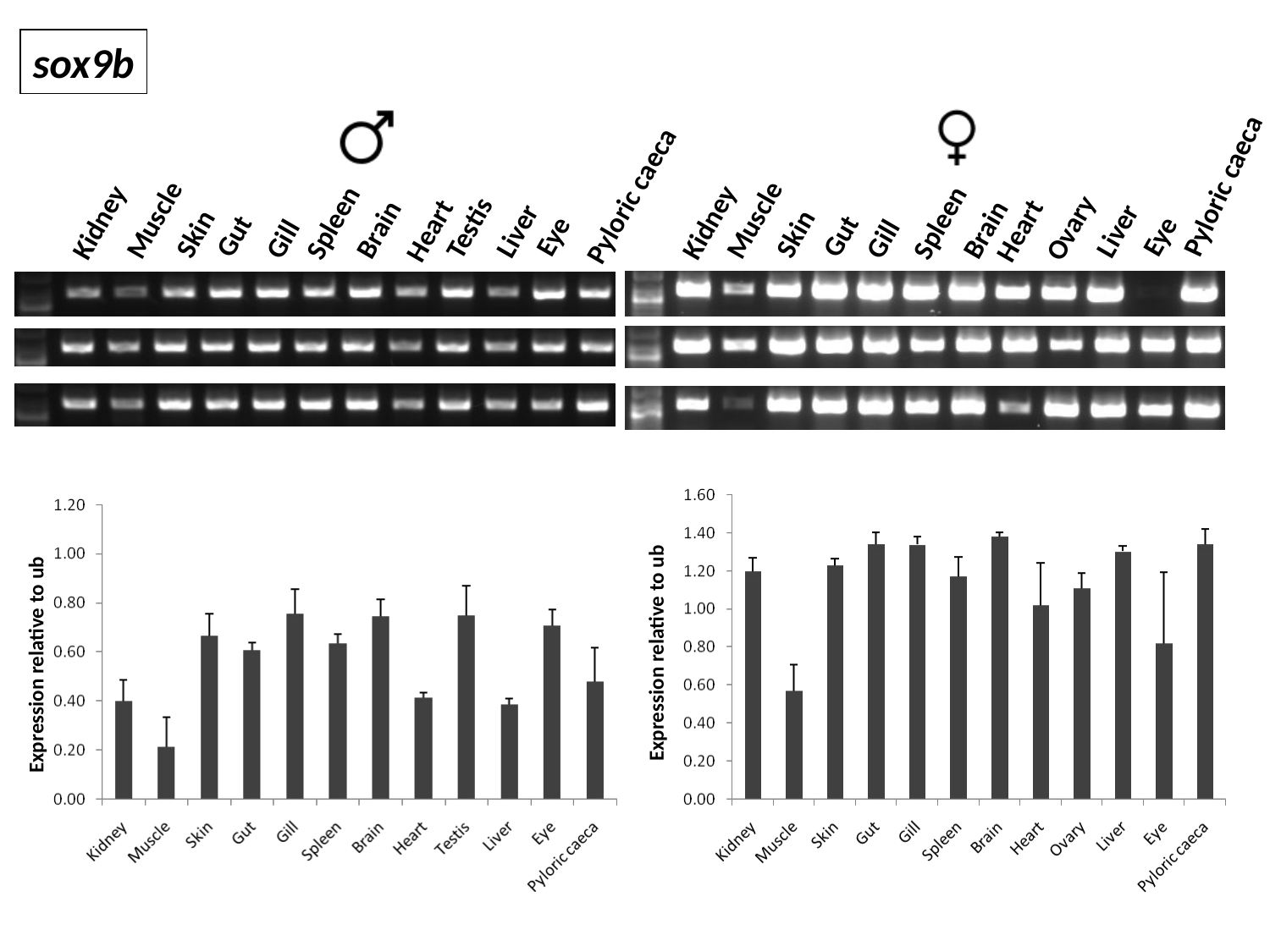

sox9b
Pyloric caeca
Muscle
Kidney
Spleen
Ovary
Brain
Liver
Heart
Skin
Gut
Eye
Gill
Pyloric caeca
Muscle
Kidney
Spleen
Testis
Brain
Liver
Heart
Skin
Gut
Eye
Gill
Expression relative to ub
Expression relative to ub
