# Supplementary material for: Regulation and expression of sexual differentiation factors in embryonic and extragonadal tissues of Atlantic salmon
Source: BMC Genomics. 2011 Jan 13;12:31. doi: 10.1186/1471-2164-12-31 (PMC3034696; doi:10.1186/1471-2164-12-31)
Supplement: Additional file 3 — Alignment of partial mis transcripts. Sequence alignment of mis cDNAs and amino acid residues based on sequence upstream from the C-terminal hormone portion of salmon MIS. A. The stop codon for mis (629 bp) is highlighted in red. The open reading frame of mis (396 bp) cDNA is in-frame for encoding the bioactive hormone portion of MIS and does not contain a stop codon. Two potential protease recognition motifs (RGQR AND RATR) for translated MIS (396 bp) are boxed. B. The stop codons for mis (629 bp) and mis (436 bp) are highlighted in red. Putative cleavage RLRR recognition sites are boxed. [file 1471-2164-12-31-S3.PDF]

**A.**

```
mis (629 bp)        TGCTTCGGTCCCTCTGTACTCTCTGGACTCTCTTCCCCCCCTGTCCCTTG
mis (396 bp)        TGCTTCGGTCCCTCTGTACTCTCTGGACTCTCTTCCCCCCCTGTCCCTTG
                    **************************************************

mis (629 bp)        GGGTGTCGTCCAGTGAGACGCTCCTTGCCAGGTTGCTCAACTCCTCAGCT
mis (396 bp)        GGGTGTCGTCCAGTGAGACGCTCCTTGCCAGGTTGCTCAACTCCTCAGCT
                    **************************************************

mis (629 bp)        CCCACCCTGTTCTCTTTCCCCACACAGGGCTCTGTGCTCCAGGGGCATCA
mis (396 bp)        CCCACCCTGTTCTCTTTCCCCACACAGGGCTCTGTGCTCCAGGGGCATCA
                    **************************************************

mis (629 bp)        CGGGGAGCTGTCCCTGCAGCCCGCCCTACTGGAGGTGCTCAGGCAGAGGC
mis (396 bp)        CGGGGAGCTGTCCCTGCAGCCCGCCCTACTGGAGGTGCTCAGGCAGAGGC
                    **************************************************

mis (629 bp)        TGGAGGAGGTTCTGGTTCAGATGAGGGCGGAGGAGGTGGGCAAAGCAGGG
mis (396 bp)        TGGAGGAGGTTCTGGTTCAGATG---------------------------
                    ***********************

mis (629 bp)        ATGGACAGACTGAGGAGACTCCAGGAACTCAGTGTCCTTCCTAAAGAGAG
mis (396 bp)        --------------------------------------------------

mis (629 bp)        CGAGGAAGCACCAGCAGGTGAGCAGGCCTCAATGTAAATTCATGATAAAT
mis (396 bp)        --------------------------------------------------

mis (629 bp)        GTCTGGGCATTAATTTAATTGCTAAGTCATTTAAACCTTTTTTTATTTCA
mis (396 bp)        --------------------------------------------------

mis (629 bp)        TTCACATAGGTGTTGGGAGCCCCAGTGAGACGCAGTACCGAGCCCTGCTT
mis (396 bp)        --------------------------------------------------

mis (629 bp)        CTGCTGAAGGCCCTGCAGACAGTAGTGGGAGCCTGGGATGTGGAGAGGGG
mis (396 bp)        ------AAGGCCCTGCAGACAGTAGTGGGAGCCTGGGATGTGGAGAGGGG
                          ********************************************

mis (629 bp)        GCAGCGGGCCACCAGAGCTGGCCAGAAGGACCCAGGGAACCAGCACCTTT
mis (396 bp)        GCAGCGGGCCACCAGAGCTGGCCAGAAGGACCCAGGGAACCAGCACCTTT
                    **************************************************

mis (629 bp)        GTCGGCTGCACAGTCTCACCGTGTCCCTGGAGAAATACCTGCTGTCTCCT
mis (396 bp)        GTCGGCTGCACAGTCTCACCGTGTCCCTGGAGAAATACCTGCTGTCTCCT


MIS (629 bp)        ASVPLYSLDSLPPLSLGVSSSETLLARLLNSSAPTLFSFPTQGSVLQGHHGELSLQPALL
MIS (396 bp)        ASVPLYSLDSLPPLSLGVSSSETLLARLLNSSAPTLFSFPTQGSVLQGHHGELSLQPALL
                    ************************************************************

MIS (629 bp)        EVLRQRLEEVLVQMRAEEVGKAGMDRLRRLQELSVLPKESEEAPAGEQASM---------
MIS (396 bp)        EVLRQRLEEVLVQMKALQTVVGAWDVERGQRATRAGQK-DPGNQHLCRLHSLTVSLEKYL
                    ************** *        *  *         *

MIS (629 bp)        ------------
MIS (396 bp)        LSPPGATIYNCQ
```

**B.**

```
mis (629 bp)          TGCTTCGGTCCCTCTGTACTCTCTGGACTCTCTTCCCCCCCTGTCCCTTG
mis (436 bp)          TGCTTCGGTCCCTCTGTACTCTCTGGACTCTCTTCCCCCCCTGTCCCTTG
                      **************************************************

mis (629 bp)          GGGTGTCGTCCAGTGAGACGCTCCTTGCCAGGTTGCTCAACTCCTCAGCT
mis (436 bp)          GGGTGTCGTCCAGTGAGACGCTCCTTGCCAGGTTGCTCAACTCCTCAGCT
                      **************************************************

mis (629 bp)          CCCACCCTGTTCTCTTTCCCCACACAGGGCTCTGTGCTCCAGGGGCATCA
mis (436 bp)          CCCACCCTGTTCTCTTTCCCCACACAGGGCTCTGTGCTCCAGGGGCATCA
                      **************************************************

mis (629 bp)          CGGGGAGCTGTCCCTGCAGCCCGCCCTACTGGAGGTGCTCAGGCAGAGGC
mis (436 bp)          CGGGGAGCTGTCCCTGCAGCCCGCCCTACTGGAGGTGCTCAGGCAGAGGC
                      **************************************************

mis (629 bp)          TGGAGGAGGTTCTGGTTCAGATGAGGGCGGAGGAGGTGGGCAAAGCAGGG
mis (436 bp)          TGGAGGAGGTTCTGGTTCAGATGAGGGCGGAGGAGGTGGGCAAAGCAGGG
                      **************************************************

mis (629 bp)          ATGGACAGACTGAGGAGACTCCAGGAACTCAGTGTCCTTCCTAAAGAGAG
mis (436 bp)          ATGGACAGACTGAGGAGACTCCAGGAACTCAGTGTCCTTCCTAAAGAGGG
                      **************************************************

mis (629 bp)          CGAGGAAGCACCAGCAGGTGAGCAGGCCTCAATGTAAATTCATGATAAAT
mis (436 bp)          CGAGGAAGCACCAGCAG---------------------------------
                      *****************

mis (629 bp)          GTCTGGGCATTAATTTAATTGCTAAGTCATTTAAACCTTTTTTTATTTCA
mis (436 bp)          --------------------------------------------------

mis (629 bp)          TTCACATAGGTGTTGGGAGCCCCAGTGAGACGCAGTACCGAGCCCTGCTT
mis (436 bp)          ---------GTGTTGGGAGCCCCAGTGAGACGCAGTACC-----------
                               ******************************

mis (629 bp)          CTGCTGAAGGCCCTGCAGACAGTAGTGGGAGCCTGGGATGTGGAGAGGGG
mis (436 bp)          --------------------------------------------------

mis (629 bp)          GCAGCGGGCCACCAGAGCTGGCCAGAAGGACCCAGGGAACCAGCACCTTT
mis (436 bp)          ----------------------------------------CAGCACCTTT
                                                              **********

mis (629 bp)          GTCGGCTGCACAGTCTCACCGTGTCCCTGGAGAAATACCTGCTGTCTCCT
mis (436 bp)          GTCGGCTGCACAGTCTCACCGTGTCCCTGGAGAAATACCTGCTGTCTCCTCCTGA


MIS (629 bp)     ASVPLYSLDSLPPLSLGVSSSETLLARLLNSSAPTLFSFPTQGSVLQGHHGELSLQPALL
MIS (436 bp)     ASVPLYSLDSLPPLSLGVSSSETLLARLLNSSAPTLFSFPTQGSVLQGHHGELSLQPALL
                 ************************************************************

MIS (629 bp)     EVLRQRLEEVLVQMRAEEVGKAGMDRLRRLQELSVLPKESEEAPAGEQASM---------
MIS (436 bp)     EVLRQRLEEVLVQMRAEEVGKAGMDRLRRLQELSVLPKEGEEAPAGVGSPSETQYPAPLS
                 *************************************** ******

MIS (629 bp)     ----------------
MIS (436 bp)     AAQSHRVPGEIPAVSS
```
