# Supplementary material for: Regulation and expression of sexual differentiation factors in embryonic and extragonadal tissues of Atlantic salmon
Source: BMC Genomics. 2011 Jan 13;12:31. doi: 10.1186/1471-2164-12-31 (PMC3034696; doi:10.1186/1471-2164-12-31)
Supplement: Additional file 4 — Proximal promoter sequences of the Atlantic salmon dax1 gene. The potential binding elements of various transcription factors, TATA boxes, exon 1 and initiator methionine codon are labeled. [file 1471-2164-12-31-S4.DOC]

CTCTTTTCTCTCTCTCTCTTTTTCGTTCTCTCCCTCTCTTCAGTAGTCAG

TAGTCTTGTACTGTGATATACAATCTGAAAGAAAAACATTCATTCTCCCT

GAATCTTGCCTCCCCCTCCTCCTTTACCTTGCCCTCCCATGCCCCTTCCT

CCAGGTTAGTCCCCCCCCCCTCCCCCACACTTCCCCGGTGACAGATGCGC

GGGGAGCGTTGAATTATGATGAGTCATGACATCATAATTCATGCGTTCCC

GTTTTCACGGTTAAATCAATAACAGTGTGTCCGACTCAAGGTTTGCGGCC

GGCCTTCACAGCGCTGCTCTCCATCCTCTTTTCTCATAGAAAATCAGATA

AATTCTCCCTAAAATAATTAAGCCTTCAGGTCCGTCTCTGGCTTACTTTT

TTACCGCTAGCGGCTGATTAACAGTGCAGTAAGGGAGTCAAGGTAAAACA

GAATAGAGCACAGGACTGATTCTTTGTCTCTCTCGCTCTCTCACAGACAA

ACACATATGCACACACATTCACATGTTTGTTTTCCTATTCTTGTGGGGAC

CAAACAATTGCTTCCCATTCAAAATCCTACTTTCCCTAACCCTTAACTTA

**IER**

ACCCCAAACTCTAAGCCTAAAATAGTGTTATTTCCTTGTGGGGACCGGCA

AAATGCCCCCATTTGTCCGAATTTTCTTTGTTGTACTATCCTCGTAAGGA

CTCCCCAGAAGGATAGTAAAACCAAAAGCGCACACATGGATCATTTTGTA

TCTTTTGGGATACCGTTGTGTCTAAAAAGAACTGGATAGGCTGATCTATC

CCCTCCCCTCCCTCACTTTTTGTGCTTTCCCTTTCCTGCACAGGCTATTT

TATCATTACTGAAGAGGTGATGCTGCTTAAAGGTGCAGACAAACTGCATT

ACATGAGGTTTGACATAAACCAACATGATGTTTTATGCTTAATAATGGAA

**GAGA factor**

TGACCTTACTATTCATCTTGCTGGAGAGAGAGAGATCACCGCTAACAACT

TAGGCTATGTTGCAAATAAGGGAAAGGGTCAGTTGTGCAACTGAAAAGTG

**OCT3/4**

TCTTTGCATTTAACCCAACCCCTCTGAATCAGAGAGGTGCTGCCATAATC

**IER or Sp-1**

AACATCCACAGCACCCGGGGAACAGTGGGTTAACTGCCTTGCTCAGTGGC

AGAAGAACAGATTCTTACCTTGTCAGCTCAGGGATTTGATCCAGCAACCT

**FOXL2**

TTCAGTTTTTGGCCCAACGCTCTAACCGCTAGGCTACCTGCCACCCACAA

ATAAGTGCCTGTCATGTTCCCCTGAATCCTACTAACGTTTACAGTTGGTG

GCATCAGCCCATGATTTGGTCAGTAATCTTCTTATAACGTATTTCATAGT

GTCTAAACTTTTGACTGGTACTGTACATCTTTATGTGCACTAACTATTAT

CATAGGCTAAATCATTTGGGGTTCAACACCTGAGGAAGTGGAAACAGTAA

ATGTAATGTCCTAAAAATACTTTGCAGTTGTAGGCTACTACTCATGAGAC

**IER or Smad**

CTATAGGTCTACTCGGAATGGCCGGTGCGCATGTCGCGAGCCGCTCCATA

TTTAAGCCATATCATATCTTGGTTGTAGAGTAGTTGCTATTAAGCTAAAA

AAAAAAAAATTAAATACCTACAGACAGCTGAGACTCTACTTACTCTCTTC

GACAGGGACAAGGGGACGCTTCCAAAGCTGTGTTTTTCCAGAAATGTAAT

**IER**

TTTGAAATGTTATACGATCAGAACAGATTTTTCTCTGGAGCACAAGGGGG

AGAGGAGATTGGCTCGCTCACCACAGCCAAACAAGCACAGGAGCAAGGCA

GACACATTAATTACAGGAATCATGAGGATAATACGCTTTACTCAATGACC

AAATCATGGTTGTAGCCACCTCAAAAATAGGATGTTTTGAGTGAGGTGAC

**FOXL2** **PPARE**

AATGTAGAATGTGCTCTTTCTACGTAAATAGGCACCCTTTTTCTTTTTTT

**SOX-like Footprint**

GGCTGTCTTTCATTTTTTATTGAACCTTTATTTAACTAGGCAAATCAGTT

AAGAACAAATTCCTATTTACAATGACGGCCTACCGCTGCCAAACCCTCCC

**IER**

CTAACGACGCTGAGACAATTGTGCGCTGCCCTATGGGACTCCCAATCACA

CCCGGTTGTGATACAGCCCGGGATCAAACCAGGGTCTGTAGTGACGCCTG

**Sp-1**

TAGCACTGAAATGCAGTGCCTTAGACCGCTGCCCCACTCGGGAGCCCTAA

CTGATGACAGAGTCGGAGCAGGTCTCTTTACTGATGCAGCGTTTGACTTT

**ERE or IER**

GAATGTGAGTTTTCGGGAACTCAGCACAGCCTTTCAGAAAACTGGGCCTA

CCTGCCCTCCAGGACACCTACAGCACCCAATATCACAAGAAGGCAAAAAG

ATCATCAAGGACAACAACCACCCGAGCCACTGCCTGTTCACCCCGCTACC

**SOX**

ATCCAGAAGGCGAGGTCAGTACAGGTGCATCAAAGCTGGGACTGAGAGAC

**SF-1**

TGAAAAACAGCTTCTATC**TCAAGGCCA**TCAGACTGTTAAACAGCCATCAC

TAATATAAGGAGGCTGCTGCCCACATAGACTCAAATCTCTGGCCACTTTA

ATAAATGTATTAATTAAGGTATCACTACTCACTT**TAAATAA**CACCTCTT**T**

**AATAA**TGTCTACATATCCTACACTACTCATCTCATATGTATATACTGTAT

TCTATACCATCTACTGCATCTTGCTATGCCGCACGGCCATCGCTCATCCA

TATATTTATATGTACATATTCTTATTCAGCTCTTCACATTTGTGTG**TATA**

**A**GGGAGTTGTTGTGAATTTGTTAGATTAGTTGTTAGATATTACTGCATTG

TCGGAACTAGAAGCACAAGCATTTCGCTACACTTACATTAACATCTGTTA

**½ ERE ERE or IER**

ACAATGTGTATGTGACCACTACAATTTGATTTTATTTGGCCGTTGCCCAC

TGTATAATAGAGAAATTGTGCATTTAAAAAAAAAAATGTATCTTATCATG

GGCACTTTTGCATTATTTTTTTATTAAAAATTATTTTTGTGTGTCCATTT

CGACCAGCCCACGAAACTAAACAAATGTTCAGCCCAACTGGCATTTGCCA

**IER or Sp-1**

GTATTGCTAGATGGCCAATCCGCCCCTGGTGGGAGGCGTCTCAGAGGAGG

AATATGCTCAAGTTTAAAGAAGTTCTCAAAGCAGGAGGTCAGACGGGAGC

**IER (WT-1)**

**TAATATAAA**TGTGCTGCCGTTTGCGCGGGGGTAACAGGGGCCAAGAAAAC

ATATCCCTCTCTATCTAGCTCTGACCTCCAACTCTTCAAAGCGCCTGACA

GACACACGGAGCAGCACCCGATGTAGCGCGCGCCACGTGCCATGGCCACT

CGCGAGGGCTGCCACTGTCCCGGCGCCGGGGGGCAAAACAATAATAACAG

CATCCTGTACCATATACTGAAGAACGACAGACTCACGACCATCGAGGAAC

AAACAACACCAAAACCACAACAACACCAACACCAACAGCATCGCTGGCAC
