# Supplementary material for: Regulation and expression of sexual differentiation factors in embryonic and extragonadal tissues of Atlantic salmon
Source: BMC Genomics. 2011 Jan 13;12:31. doi: 10.1186/1471-2164-12-31 (PMC3034696; doi:10.1186/1471-2164-12-31)
Supplement: Additional file 5 — Proximal promoter sequences of the Atlantic salmon dax2 gene. The potential binding elements of various transcription factors, TATA boxes, exon 1 and initiator methionine codon are labeled. [file 1471-2164-12-31-S5.DOC]

ACAAAATGTGGACAAAGTCAAGGGGTCTGAATACTTTCCGAATGCACTGT

ATGTGAGCACACATTCATAGGCCTTCATAAATGCATATAACATAACCTGT

CCCTATTGCACTTGTTTGTATCTGAAGACCTCTTCGTCTCTGTGTGTCCC

AGAGAGAGTGCACTAAAGAGACACTCCAATAAGCCCTCTATTGATGTGTG

CCTGGTGCCCCTCCCCCTAAAGACATCTGTAGCAGCTGCCCCCTTGTACT

CCTGTCAGCAGGGCAGGGGGTGGAGTGGTGTGTGGAGGGGTTAGAGGGGG

**IER**

CGTGTTAGGGCACCTCCCCTTTGGGAGGGGGCCAACCCGCACACTTGTGC

AACATCACATTCATGAATGGCTAATAGTCGCTATCAGGTCACCCTGAAAA

CACAACATCGACCTCTGACCCCTTTTTAGCAAAAAGCCGGTACACTGGTC

**CT-rich block**

ATACTAGCCCCTGATGAATCTTCTCTCTCTCTCTCTCTCTCTCACACACA

**CA-rich region**

CACACACACACACACACACACACACACACACACACACACACACACACACA

CACACACACACACACACACACACACACACACACACACACACACACACAGA

**ERE**

GGGAGTAGTTGTGGGTGGGTAGTACAGGGGTATGCTGACCCATGTTTTAC

**ERE or IER**

TGGGCAGGGGACCAGCAGATCTGGGCCCAGAGAGACAGTCTGGATCACAT

CACACACAGACAGGAGAGGACAGGAGAGGACACACTGGACCTAAGAGGGT

**CT-rich region**

GATATCACTGCCTCTCCCATCTCCTCTCTCCTTTCCTTTCCTTCCCTCCT

CCTCTTCTTTTCTCTCCTTACCCATGCCCTCCACTCCTCTCACCCACCCT

CTGCTCTCCCACCCTCTCTTTCCCTCTCCTTTCACAACCCCTTTCCCCGC

CTCCCCTCCATACCTCTCCCGCTAAGCCCTGCCCAGCCATAGCCCAGCCA

AAGTATGAGATCATGATTATACATCTAAGTCTATAAATCTGCTGCCAATC

TGACACAGTGGCTTTAACTGTTAAGTGACATTAACAGCTTCCAACCCAGC

ACTTTTCTGTTTTGTACTCTTACTCATATGAACATTCAAAACAGTACAGT

AAAACGTGAGTGCCTATCTGTCTTTGCAGTGGGTGGAAAATGATTGGCCC

TTTTCTGTGGCATTAAAGAAACAGACTTCTGAGGCAAAATCACAATTCAT

**IER**

GGGTAGGGGCCTGAATTCGCTTTCCATCCTCATTACCATAAACAGATTAA

CAAGTAGAGGAATAGAAGACTATGACAGAATGAGAAGAATTTGTATTACC

TGAGAGGCAAGGAGAGCAGAGGAGAGTGTTGGAGAGGCGAGGATTTCAGA

**GA-rich block**

GGAGAGGAGAGGAGAGGAGGAGAGGGTTGGAAAGTAGCAGAGGATAAAGT

TGGAGAGGAGAGGATTGGAGAGGGTTGGAGAGGAGAGGGTTGGAGAGGAG

GATAGGGTTGGATAGGTGGAGAGGAGAGGGTTGGAGGAGAGGGATGGAGA

GGAGAGGTTTGGAGAGGGTTGGAAAAGAGGAGAGGATTGGAGAGGAGGAG

AGGGTTGGAGAGGATTGAAGAGAGGAGAGAGTTGGAGGGGAGAGGGTTGG

**GA-rich region**

AGAAGAGGAGAGGGTTGAGAGGAGGAGAGGGTTGGAGAGGAGAAGAGGGT

TGGAGAGGAGGAAAGGGTTGGAGAGGGTTGGAGAGGAGGAGAGGGTTGAG

AGGAGGAGAGGGTTGGAGAGGAGAAGATGGTTGGAGAGGAGGAAGGGTTG

GAGAGGGTTGGAGAGGAGGAGGGGGTTGGAGAGGGTTTGAGAGGGCTGGA

GAGGAGAGGGGAGGGTTGGAGAGGAGAGGGTTTTAGAGGGTTGGAGAGGA

GAGGGTTGGAGAGGAGAGGGTTTTAGAGGGTTGGAGAGGAGAGGGGAGGG

TTGGAGAGGAGAGGGTTTTAGAGGGTTGGAGAGGAGAGGGGAGGGTTGGA

GAGGGTTTTAGAGGGATGGAGAGGAGAGGGGAGGGTTGGAGAGGGTTGGA

GATGAGAGGAGAGGGGAGGAGACAGAGTTCGGTCTTGTTCTTTATCCCAG

**FOXL2**

CTCGACCTTGAATATAAACATGTAAAACATCTCTATTTTCTTGCTGCTGT

GTATCAATAGAAAGCCATTCTCTCTCTGTTTAATTTTTATTTATTTATTT

TATTTCACCTTTATTTAACCAGGTAGGCAAGTTGAGAACAAGTTTTCATT

TACAATTGCAACAACACAGAGTTACACATGGAGTAAAACAAACATACAGT

**SOX**

CAATAATACAGTAGAAAAATAAGTCTATATACAATGTAAGCAAAAGAGGT

GAGATAAGGGAGGTAAAGGCAAAAAAGGCCATGGTGGTGAAGTAAATACA

ATATAGCAAGTAAAACATGTTCTGAAGCTGCTGTCAAAGTTTCTCTTCCT

GTCTTGGGTTCTTGGAGCACACAGAGTAGAATGAGGTCTCTCTCCCCTGT

**SOX** **SF-1**

TTATGAATACAGCTAGTCACTCAATGCCAGTCATTTCT**CCAAGGCCA**AAA

ACACCTTTTCTAAACACTATATAGTAGAGGCAGTGTGGAGGTCTTTGTTC

AGGCTCTCCCCTGGACTACCAAATGTGCTAACACACTCTCAAGGCTCATG

CACTTACAACCTTTCCTATACTTGATGCCCCATATGTGTCTTTCTCTCTG

**CT-rich regions**

TCCTTCTCTCTATCTATCTCTCCCTCCCTCTCTCCCTACCTTCCTTCCTC

CAGGTTAGTCCACCCCCCACCCCACCCCTGGTGACAGATGTGCAGGGAGC

GTTGAGTCATGACATCATAATTCAAGCGTTCCAGTTTTCATAGTTAAATC

AATAACAGTGTGTCCGAGTCAAGGTTAACGCCAGGCCTTCCCAGCGCTGC

TCTCTCTGCTCCTTTCACATAGAAAATCAGATAAATTCTCCC**TAAAATAA**

TTAAGCCTTCAGCTCTGTCTCTGGCCTATTTTTTACCGGCTAACAGCTGA

TTAACGGTGCTGTGAGGGAGTCAAGGCAAAATCCAAAAAATATAGTAGCT

**CACA box**

CTCTCACACACACAAATACAAGTGTGCACACACAGACACACACACTCATT

CACAGACATAGAGTAGGTATGGATATTTGTTTTGCTTTTGGGATAGTGAG

ATAAATATATAGACCGTTGTGTCCAAAAAGAACTGGATAACCTCAAGGGT

ATAGAATCAGTCAGTCTGTCTGCCCCCTCAGTCTCTCTGTCTGCCTCCTT

GTGTGATATAATTGGTATACCCTGAGGTACAGTTTGTCCTTTTCCTGTAT

AGGCTATTTTAAACTAACTGAAGGAGTGATGCTGCTTAACATGTTATTTT

**½ ERE**

ATGATACTGGAATGACCTTACTATTCATCTTGCAGCAGCTGGCTCCCTCT

GGGTGCATGTGCTGGGGAGGACTGGAGCAGCACAGGGAGAAGGGGGGGGG

**GAGA box**

GGGGGAGAGAGAGAGAGAGCATGAGTGAGTGAGTGAGTGAGTGAGTGAGT

GAGTGGGAGGCGTCTCTGAGGTGGAATATGTAAAAAGTCTAAACAACAAG

TCCTCAAAGCTCGAGGACAGACGGGAGCTAGTATAAATGTGCTGCTGTTC

GCGCGGGGGTAATAGGGGACAAAAACCACCCTCCCCCTCTCTGGATGTAG

CGCGCGCCACCGGCCATGGCCACTCTGGAGGGCTGTCACTGTCAGGGTGC

CGGCGGGCAAAACAATAACAACAGCATCCTGTACAACATACTGAAGAACG
