# Supplementary material for: Regulation and expression of sexual differentiation factors in embryonic and extragonadal tissues of Atlantic salmon
Source: BMC Genomics. 2011 Jan 13;12:31. doi: 10.1186/1471-2164-12-31 (PMC3034696; doi:10.1186/1471-2164-12-31)
Supplement: Additional file 6 — Proximal promoter sequences of the Atlantic salmon mis gene. The potential binding elements of various transcription factors, TATA boxes, exon 1 and initiator methionine codon are labeled. [file 1471-2164-12-31-S6.DOC]

ATACGTTTGTGTTAAGAATGGTGTGTAGGTGAATAAGAAATGCCTACATT
GATTTGGGGTATTATATTTGTAAAAAATGTTCTTTTGGTGCTGACGTGTG
ACCCACATCTACAGCCTGTCAAACAGGTTGGGCTTGGACCCAAGGGAAAC
AGCAGTGCAGAGTTGTTAAACCAGGGCCTTAATAGAGTGAGTGAAACACC
ATGACTGTTCAGTGTCAATGAGGGCATGCCAGTCACGTGGCACCGTCACA
TGGCACCATCTCCTCTGATATACACAGAGAAACTCTATGCTTGTACTATT
ATGTGGTTAACACATAGAGGGAGAGATTTGTGCTGCAACTAGACTGTCTT
GATTTGTTCTGGATTGTAATCCGATTTACTTCATGCTTTTTAAAGAGAAA
CTTCTACTCTGTGAAACAACATGCCTTTCAGTAATTGTAAAAAATAATAT
TTTAAAGTAGAAAAGACAAGGTGCTGTTTCAACCGTACATACTGATTTCA
GATAACGTTCTGGGTGCATCCAAAAGCCAAATCTCATCTTTCACTTCTGC
CTGTCCTGGGATATGTCTTAAAGCCGCCTCCTTCCCATAGTATGAGCATA
ATGCATCTGTTGTAATTACAAGTTAATTGTTCCAATATAACTGGTAGTAG
AATAACTAGTATGTGTTATGTGGGTTTGCAACAACCAGGCTCCATTTTGA
TCTGATTGTCATTTGTCAGTCCTTACTAATTGAGGACGTGAAGCAGGATA
TTAATTTATCATCGTTGTTTTGTTTGTTTCATCTGTGTCCTTGAGGAGTA
ATAAGACATCACAGCTTTATAGTCACAATGTTGTCCAGTAGACAACAGTG
CTTTGCAATCAGGAATGAGGACAATGATTTGCTCATGTCGGTCATGACCA
GAGAAGCTAGTCCCCTGTTATCAGCACTGACAGGTCTTTTGTAATTACTG
TTATTGTTGATGTTTTCATAGTTTTTTTTATTTTTATTGTTTGATATAAT
TGCAGTTAGTATCAATGATGCTTTTATTATGACATAAGATTCAGATCAAA
TTGGAATACTCTTAGACAGACAGTACGGTGCTCTCCTACACTTAAATCAA
GGAAAATGCAGCTATTGGTTAATACACTTCCTCTCCTGTTGTGCTAGCGG
AGGGAAAAGGTTTAATAAACTTCAACACAAGCTCGCTTGTTGACTTATTA
TTCATGTCCCATCTGAAAATATACTTCCTAGTTGGCTGGAGTGGCAATGT
GTGGGAGAGTGAATATTGTTACTTCAGGTTTGAACAGTTCAACGGACCCC
ATTTTAAATATGTACCCTGAAGAAGTACACATGATCTCTGGCAAATTTGG
ATACATATTTAATACTGTAGTGCAGTATACTGCTCTATTAACTTTACAGT

**½ ARE**
TAAGACTGTTTCACATAACAAGACTGAATCCTGCCAAAGGTCTCACTCAG
GAACTTAGCAGATATCCTGAAATGATATAACACTACTCTATACGTCACAC

**PPARE**
TTATCTATAAACTGTGACTTCATTATTTCTTTCCCTAACTCAGGACCAGG
GACCTCTTATATTTTGATGTAACTTAAATTCATATTGGTTCTTGGAGTTG
ACAAACTTCCTCGAAAATGTACGTTTGTCTCCTCAATTTCTCAGTCGCAC
ACATCCAGCCACTCCAGCAAAAGATTGCAGCTTTGTGTTCGACTGGTCTG
ACAATGTCGTGGAAAATCCTCTTCTTACACTCACAAATGCCTCGTCTCAC
TTCCTCTCTCTGAAAAGGAATTGTCCTTTTTTGTGATCCCTACATAGGGA
GAACGCTGTGTATTTGGACTGAATTTTAACTCCGTATGTGGATCAGTAAA

**FOXL2**
ACTAAGGGCAGGTTTGAGGTGCAATAACAGCAAACCTACTTTCCAAAACG

**SOX-like Footprints**
CCTTGGGCTGTCATGGGAACTCTTGGTGTTGGTGCTATTATCTTAGATGT
TTAATGACAAATTGAAATTTGTTTTAGTTTTTTTGCCTTTTTATGATAAT

**OCT3/4**

TATTAATAAAATGGTTTCTTATAAAATATTTAATTTGCATCTCTGCTTAT

**OCT3/4**
TGGTGATATTTTGCATGTTGAAAAGGTGATTTTATTTATACTTTTTATTC
AAAAAATTGTAGGATTTGTATATGGTGCATGATATTTTAGTTTGTTTTCA

**IER(WT-1)**
GTGAAGGATTCAGACTATGTAGGGGTGGGATGAGTGTCATTTTTCTAGTT

**½ ERE**
CTGGTACATTCTGTACTAAAGCTCAGCTTATCATGGTTTTGACCAAAGAT

GGACAGTAGTGTTTTGAGAATTGTTCTTCAGTTCTGTCTTTAGTGGGGAA
TACACATTTAACTCCAAAATGTCCACTATTGGGGAATAATTCAACATTCA
ATGTCATCTTTATGAATACTATCTGAAATAGTGTTCTGCAATGTTAGGTG
CATGTTTTCAAGCCATTAGAAATGATAAATTCTTGTAATTTTTAGTGGAT
GATTTGTGAATCCTTGACTTTATCACTTGGCGCTGTTGTCCACCAAGCAG
GACAGACTGAACTGAACGACCTTGATCGTGTGCAAAACAAAGCTGCAGCT

**SF-1**
GTTGGATTTCTCCATGAAA**CCAAGGCCG**TGAGGCACTTTTCAACCCGAGA
AGTCTCAACAGCAGCATCTCATAGTCTGTCCATAGATCCTTTAATCTGTC
AAGAGCTCTTATTTAGCCAACACTTCTTCTGTGTCTATCTGGCATGCAGC
ACATCCATATTCCATCCACTGTACATAGTCCAATAACTGCTGAGAGAACT
TTTTAAAGCCAACGTGTCTGTAATCAATCGTCACTTTGCAATCGCATCCT
TAGAATTGCATCACAAATGGCTACTTTTATGAGGAACGTTCCAATATCTG

**IER(WT-1)or SP-1-related**
TTTGTGTATTGGGGGGGAAAATGACATCTGGTGGGCCGAATTTGGCCCCC

AAGTTTCCTAAGCATTTTCTTGTATTTATTATTTTAGAAATC**TATAAA**GT

**FOXL2**
ATTCCTACGCAAGGTGTGAAATTATCATGTTTTAGTCAAATATACCTGTT
TGGGCTTCTTGCGTTCAATTTGCAGTCTACAAATTATTTGTAATTTCTGT
ATATTCTGGCCCCTTGACCATCCGCTCAAGAAAAAAAAAGTCTCGCCGCT
GAATCTAGTTGATCATCCCTGAAGAAGAACTGTATGTAAGAAATGGCAGG
ACACAGAAGCAATAAGATAGTGTTGATATGCTGTAATAGACCAGACGTTA
CCTATGAAAGGCCTTTATAGAAATGTCATTACACGCATGGTTTTTCTATC

**SOX-like Footprint**
TCATTCAAACATCCTCCCTTCCTTTTTTGAGGTGCTTCAATCTCCAGTCT

**GAGA factor**
TTTCTCACCCACAGAGAGGGCAGGTGTCTGTCTGCCACACACAAAACACT

**PPARE**
CCATTGTCTGTCTTGTGCTTCCTTTCCCTAGCCTTATCTCAACCCACCGT
AGTATTTGCAGCATTCACTGTCCCTCCACATCTTACAACATAGGATAGTT
TCTCAAGGACACATCACATAGTATTAGCTGCGTATTTGGCAGCCTGTCCC
CCTTAGCTGAAAGAGGGGCGGTGTACTACATAAAACCGATCTCCCTCGCT

**Exon 1**
GACCTGACCCAAAACAAGTGTTCGATCCAGACGTGACTGGACAGACAGGG

TTACCATGAGGCTATGGTGCATATTCGGCTTGATACTGCTGCTGCCCAGC

ACAATGGTCACTCTGCCACACCAAGGCAGACTGAGTGACAGCCTGCTAGG
