# Supplementary material for: Regulation and expression of sexual differentiation factors in embryonic and extragonadal tissues of Atlantic salmon
Source: BMC Genomics. 2011 Jan 13;12:31. doi: 10.1186/1471-2164-12-31 (PMC3034696; doi:10.1186/1471-2164-12-31)
Supplement: Additional file 7 — Proximal promoter sequences of the Atlantic salmon foxl2b gene. The potential binding elements of various transcription factors, TATA boxes, exon 1 and initiator methionine codon are labeled. [file 1471-2164-12-31-S7.DOC]

ATTTGTACATCCAAATCATGTTACATGCACTAAGCCTGATCTTTCTTTTG

GTTTTGGCACTATCCTGTGCTGCATTCTTGTTTGGTCTCAAATGGCTTAG

GCTGCTGCATGGTAGTTCTGTAGAGATATTGTATTTTATCTGGCGCCTTG

CTTTGCTCTCATGACATCAGTCAGTAAATCTAGCATGGGGAGACTTTCTG

AGCTCCCTTTAATAACATTATCTCTGATAGAATGTAAGAATGTAGAGATT

GCAGTCTGAGGGCATGGGTGATTTTACAGAATTGTACCTTATTTCACTTC

**OCT4**

CTTCTGTTAAGCAAATAGATGTACCCAGTAGAATTATGAGGTAGCCTACA

TATTTTTGAGATACTGTAAAACTTTCTTCAGTTTGGGTTTGTTGTTAAAT

GTATCAATGTACCACTAGCTATTACATCTGTATCAACTGTCCAAATGGTC

ATCCTGCTACAATGCCTTGAGTCAGCTAGTTTGAGCTGAAATCAAGCGTC

ACACAGGTGCCTTTATACAGTGAGCTATTTCACTCAGAGATCTGTCTGTT

TCCCTATCAGTCACTCAGGGGAGACAGTCTGTCTCCTGAGAGCAGGTGAT

CATTATCACCTGTCTCCCACAGGTCCGCTGGTCATGCCTCACTGTATGTG

GGTGGTGGCATAAAAATAAAATGACTAGAACGAGTAAGCCCTTCACACCA

CAGCAACTTGACTGTAGGTACACTCAATATGGCTGAAGGTCCACCGATCA

CGGAACATCGACTTGAATGGGAATGTTTGTTCTGGTCATTCTATTTCTAT

GGGTAGCAGTGATTACGGTCTCTACAATGTCTATGGTGTCTACGCCTGGC

CATGTTGTATTACTCAGCATTTCTCGCCCGTGTCTCTCCACCACCAAGGG

CATTAGCCTATTATAGAAGCATTTATTCACAAACCTCAAATTCAAACAAT

TACTGTCAATTAATTTCATTTCTAACCTAAGTGGGATGGTATTTTAGATC

AAGGCCTATTCAAATTATAGTTATTTTTATTGCTTTGGTGCTATTTTCAC

**OCT4**

AAGTATCTGGAAACTCTTAGTACAAAACTCAAAGCAAATCATCAAAAAGG

CTGTTTTTTCAAAATTTTAAGCACATTTTCAATTGATATACACAATCACA

CAGTAATTTTTTCACCAAACCTAATCCGTGTTTCATCTATATAGAAATAC

CTTTCATATAAAGCAATTGCCTTTCACAATGCAACGCTCACAATACTTTC

ATATGATTCTCTTCTCATTTGTTTAAACCCAAATGACATGCATGATACAT

GATAACCATACTTAAGGACTACTCGTTATTCCTAATTGATTTCACATAGC

GGTAACCACAATTGGTCACCATATAAAAGGGTGAGTGTGAACAAGGGAGA

GGAAGAAACCAAAGAGCTCGTAGACAAGGGACCCGTCAGAGAGGTGGGCA

TGGGCCCCGTCACTACTTGTATTTGTGAGAGGTATTGACATGTTGAATGC

ACCGAGCTGTCTGCTTGAACTACTGGCACACAGCTCGGACACCCATGTAT

ACCCCACAAGACATGCCACAAGAGGTCTCTTTACAGTCCCCAAGTCCAGA ACAGACTATGAGATGCACACAGTACTACATAGAGCCATGACTACATGGAA

CTCTATTCCACATCAAGAAACTCATACCAGCAGTACAATTAGATTTAAAT

CAGATAAAAATACACCTTATGGAACAGCGGGGACTGTGAAGCAACACAAA

**CACA box**

CATAGACACATGCATACAAACACACGATAACATACGCACTATACACACAC ACACACACGTACTCATGGATTTTGTGTTACAGATAAGTGGTAGTAGAGTA

**½ ERE or RXR/RAR**

GAGGCTTGAGGGCACACACTTAATATGTTGTGAAATCTGTTATTTAAAAT

**½ ERE or Egr-1**

GTATAACTGCCTTAATTTTGCTGGGCCCCAGGAAGAGTGGCTGCAGCTAA

**FOXL2** **GAGA factor**

TGGGGATCCATAATAAATACAAATACAAAGAGGTGGACATCAAAGAGAGG

**Sp-1** GAGACAGATGGCAGTGAACTGTTGTGTCTAATGAAGTCCGGGCCATCATC

AATGACCATGTGGTAAACAGAGGCCTTTACCATGGCAGACGCTGCCAGAT

**IER**

TAGTTCACCCCAATGTGAAAAGGTCAGAAGTGGTTCAGGCATGGTTTCGG

GCCCATCCTCCATCCGTGACCCTATACCTGCCCCCCCCTACTCTCCTTTT

**CRE** **SOX**  TTCCCCCAGCAAAGGAGCAAATATGGTATCACGTCACCAATGGTTTTACA

ATGTTCTGCCATGAAATAGTCCAGAGAATACTTCTACTTACAGGTCTGGA

TGGAATGGAAATGTCTATGTATGAATAAATACAGAAAAATAATACAGGTA

**IER(WT-1) Smads**

GAGGTGTGTGTGGGTGTGTGCGTGCATGCATGCATGCATGCCTGTGTGTG

GGCATAAGTCAGTGTGTTTCAAAATGTGAATGTGCTATTCATGGTGAACT

GTAGAACGCACTGTGTGGATAAAAGGGAATTTCCATGTTGCAACACTACA

**ERE or RXR/RAR** GTGTCAGGCCATTAGGCCCAATCTGTCATTCAATCCATTAACTGAAAAGA TTCTTGTCATATTTCGTGTTGACATAATATTATATAGCTATGAAGTTTGT

CTTAATTGTGCTTAAAACATGTATTTCTATTTTTAAATGTCTTATTTTTC GAATTAAATTGGTGACAAAACCTGAAAAGCAAAAACTAAATGTATGATGT

**½ ERE**

GAAATGTTAATGGGATATTCGTATTTATGTCGTTGACC**TATATAAA**TGAC

AT**TATAAAA**GGATGAATATAATTTATACAATTAGGGTGGAAATATTT**TAA**

**IER ATAA**GGCTGTCTGGCCTGCATCATGTAGCCGATTGGTAGTCTATGTGTCT

CTATATTGGGGTAAAAGTGCGGTGTTATTGTAGGTCAAATAGTTCA**TATA**

**ATA**GCTTGCAGTAAGAGAAAAGTCAGATCACTCATCCCTGCTTACAAACA

TCATCGCTTGACACGTCAGTTGAACGCACTCACACAATCTATAGTTCACC

TTGTTCTATGCGTTTCAAAGGGCTTAATAGTATTGTAAAACTTCACGAAT

**½ ERE or CRE**

ATCGATAAACAGACAGACAACATGTCTGTGACCTCATTTTCAACCTAAAC

AAATAACTGTTGACCCTAAAGAACTCAGCAACAGTGTGGGAAAAGCGGCA

**IER (WT-1) ERE or HRE**

ATATGAAAGGGTGTCCCAGCTAGGGCAGACATCCCTATGGGAGGGTTTTA

AAACAGTAGCGGAATGCGCGTCTCACCTGTGAACACTATTGTCCAAAACT

TTTCCCGTGTGGTTGGCAGCTCCTAAACTGAGGAAGATTTAAACTACATG

ATAAGATGGACAAAGAGGACTTTCACGATGAGCAGCGATTGGACATATTG

GATCTAACCGACTCGTCCTCGGGCATGGCCAAGGGCTCCGCAGAGAGCAC
