# Supplementary material for: Regulation and expression of sexual differentiation factors in embryonic and extragonadal tissues of Atlantic salmon
Source: BMC Genomics. 2011 Jan 13;12:31. doi: 10.1186/1471-2164-12-31 (PMC3034696; doi:10.1186/1471-2164-12-31)
Supplement: Additional file 8 — Proximal promoter sequences of the Atlantic salmon pseudogenic foxl2a gene. Potential TATA boxes and the 5'-end portion of a region of sequence similarity shared with rainbow trout foxl2 genes are labeled. [file 1471-2164-12-31-S8.DOC]

TGTTGCCACAAAGTTGGAAGCACAGAATTGTTTAGAATGTCATTGTATGCTGTAGCATTAAGATTTCCCTTCACTGGAACTAAGGGGCCTAGCCCGAACCATGAAAACAGTCCCAGACCATTATTCCTCCTCCACCAAACTTTACAGTTGGCACTATGCATTGGGGCAGGTAGCGTTCTCCTGGCATTCGTGTGATTCATCACTCCAGAGAATGCATTTTCACTGCTCCAGAGTCCAATGGCAGCGAGCTTTACACCACTCCAGCCGACGCTTGGCATTGCGCATGGTAATCTTGGGCTTGTGTGCGGCTGCTTGGCCATGGAAACCCATTTCATGAAGCTCCCGACAAACAGTTCTTGTGCTGACGTTGCTTTCAGAGGCAGTTTGGAACTCGGTTGCAACCGAGGACAGACAATTTTAATGCGCTTAAGCACTTGGCGGTTCCGTTCGGTGAGCTTGTGTGGCCTACCACTTTGCAGCTGAGCAAAGTTTCCAATTCACAATAACAGCACTTACAGTTGACCAGGGCAGCTCTAGCAGCGCAGAAATGTGATGAACTGACTTTTTGGAAAGGTGGCATCCCTATGACGTTGCCACGTTGAAAGTCACTGAGCTTTTCAGTAAGGCCATTCTACTGCCAATGTTTGTCTATAGAGATTGCATGGCTGTGTCCTCGATTTTATACACCTAGCAGCAACAGGTATGGCTGAAATAGCCGAATCCACCAATTTGAAGGGGTGTCCACATACTTTTGTGTAGCTATATTGTATTATTTAATGGCGCTTGACGGGACAGCTGCCGTTTTATGTGCTCCAAAGCAACTGTGCTGTTTTGTTAATTTTTTCGCGTTATTTGTAACTTCGTTTTTTTTACTTATTTTGTACATACAGTAATATTGCTGCTACTGTCTCTTATGACCGAAAATAACTTCTGGACATCAGAACAGCGATTACTCACCTTGAACTAGACAAAGATTTTTTTCTTTAATGAGTCCGACGCAAAGGGTATACTACTTTCTCGGGAACTGGCCCAAATCCCCGTCATTTGCGTGAAGAAAAGATGGAGAAAAAGGGGGCGGCGGTCGGGCGAGTGAGTAAACACCCTCTACCATCAGTCCTATTAGCCAACGTACAATCATTGGATAACAGAATGGATCAGCTCTGATCAAGGATATCCTACCAATGGGACATTAAAAACTGTAATATCTTGTGTTTCACAGAGTCGTGGCTGAACGATGACATGGATAACATACAACTGAGGTAGAGTATTTCATGATAAGCTGTAGACCACACAATTTACCAAGAGAGTTTTCATCTATATTTTTTGCAGCTGTCTATTTTTCCACCACAATCTGATGCTGGCAGTAAGACGGCACTCAACGAGCTGTATACTCCACACACAGAGACGAGTACGATGCTCTCCCTCACCCTCCATTTGGCAAATCTGACCATAACTCTATCTTCCTGATACCTGCTTACAAGCAAAACTAAAGCAGGAAGCACCAGTGACTGGCTCAATAGCTACAGGACTGTTTTACTAGCACAGACTGGAATATGTTTTGGGATTCTTCCGATGGCATTGAGGAGTACACCACATCAGTCACGGGCTTCATCAATAAGTGCATCGATGGCGTCGTGCCCACAGTGACCATACGTACATACCCCAACCAGAAGCCATGGATTACAGGCAACATCTGCACTGAGCTAAAGGGCAGAGCTGCCGCTTCAAGACATCTTTCTATGCCCTCCGACAAACAATCAAACAGGCAAAGCGTCAATACAGGACTAATATTGAATCGTACTACACCGGCTCTGACGCTCGTCGGATGTGGCAGGGCTTGCAAACTACTACAGACTACAAAGGGAAGCACAGCCACGAGCTGCCCAGTGACACGAGCCTACCAGACGAGC**TAAATAA**CTTCTATGCTCACCTCGAGGCAAGCAACACAGAAGCACGCATGAGAGCATCAACTGTTCTTGACGACTGTGTGATCACTCCCTCCTTAGCCAATGTGAGTAAGGCCTTCAAACAGGTCAACATTCACAAGGCTGCAGGGCCAGATGGATTACCAGGACGTGTACTCCGAGCATGCGCTCACCAACTGTCAAGTGTCTTCACTGACATTTTCAAGCTGTCCCTGACTGAGTCTGTAATACCAACATGTTTCAAGCAGACCACCATAGTCCCTGTGCTCAAGAACACCAAGGTAACCTACTTAAATGACTACCAACCCGTAGCACTCACGTCTGTAGCCATGAAGTGCTTTGAAAGGCTGGTTATGGCTCATATCAAAACCATTATCCCAGAAACCCTAGACCCACTCCAATTTGCATACTGCCATAAGAGATTCACAGATGATGCACTCCACACTGCTCTTTCCCTACTGGACAAAAGAAACACCTACACGAGAATGCTATTCATTGACTACAGCTCAGCGTTCAACACCATAGTGCCCTCAAAGCTCATCACTAAGCTAAGGACCCTGGGACTAAACACCTCCTTCTGCAACTGGATCCAGGACTTCCTGCCAGGCCGCTCCCAGGTGGTAAGGGTAGGTAACAATACATCTGGCACTATGATCCTCAAGGGTGCATGCTCAGTCTCCTCCTGTACTCCATGTTCATTGTTTGCTATCATGTGATTCCAAATCCTGAATCAATCCATACTATTTGACCACAGATAGTGCTATCCAGAATCCTTGGCATGTCCCTACCCTAAACCTAGCCTTAACTCTTACCCTAACCATAAACCTTACCTAACCCTTACCTTAACCATTTTAAATGGGATGTCCCAAGGATCCCGGATAGCATGGGCCATTTGACTACAGGCAGTAACCTTCGCTGTTGGTTAAACAGGCAAGACTGCTAAGCAGTGCATGTAGCCCTGCCCCACGTAGAGTTGGTAGCTAAATTGAATATCCAAGTGTTCATTGGCTGAGCCGGCAAATACGTTTTGTTTTACTGGTATATGCCCAAACCACTGTTTCCACTCAGGCCAAGCTGTCTTTGACAATTTTAAATGTGACAGATTTTCTTTTTGCAAACAGTGCTGAGATCTGCAATTTACAGTTGCTAAGTACGCACTTGTTATTCTTGAACATTTTAGCACAGAAGTGATCCAGACATTTTGTCTACATTCAACGGCAATGATCAGCTACTGATTAATGATGAAAATGACAGGGATTGCAATAAC**TAATA**CAAATG**TATTAA**TTGATGCTATCTTTTTCACTGAGACAGTACACAGTTATCAACATGTCAGTTTACACGTCAATATACATTTTCCGGAGTAAGCTATGAAGCTAATTTTCATCCTTAACACCCGGGCATGCTACACAACCCACAACGTACTGAGATGCATTTTCAGATATGGTTAGATTTGTTTGCAAGCGTACTCCATTAGGATGTGAATCTACTTTATCTGTTAAAATTTCGTTTTAAAAAAACAATGTGTGTCTAAGTGTTGGAAAAATGAATATTTTGTCACACAGTAGCCTAATAGTACACCCACAATTCAAAAATAGAATGGTAGGCTTGTCTTGGGAGTGGATCAGCTGGTTGCTGTGGAGTGAGTTGCATCGCTGTGGGCTGCAGGCCAGACTGGTAGAAAAAGGCCCAAGTGCTGTCATATAGAACCTGCCCAGTGGTGCATTATGGGAAAAGACACGGGTCACTGGCTTGGTGAACTGAACAGGGAACTCTTGTATCTCATCACTTGAGAGAAGCAGGGCCACACCTAGCACAGGAATTTAGCCAAGGCAAACTGACTTGAAGAAAAACCATTAGGTCTTCTATTGAAACAAAATATCTAATCTATCATCTCTGTTTTTGGTTTTATGCTGCTTCATTATTGTATGTATGACCTAACCTAGAGAAGTCATCACATCACGACTGATGACATTTT

**start of shared similarity with trout *foxl2* genes**

CTATCCCACAATCCTTTCCTTTCTCTAGAGTTTCACATTTCCCTGCTATGAGAAAAACAAGAAGAGGTGGCAGAACAGCATTCGGCACAATCTCAGTCTC
