# Supplementary figures and images for: Regulation and expression of sexual differentiation factors in embryonic and extragonadal tissues of Atlantic salmon
Source: BMC Genomics. 2011 Jan 13;12:31. doi: 10.1186/1471-2164-12-31 (PMC3034696; doi:10.1186/1471-2164-12-31)

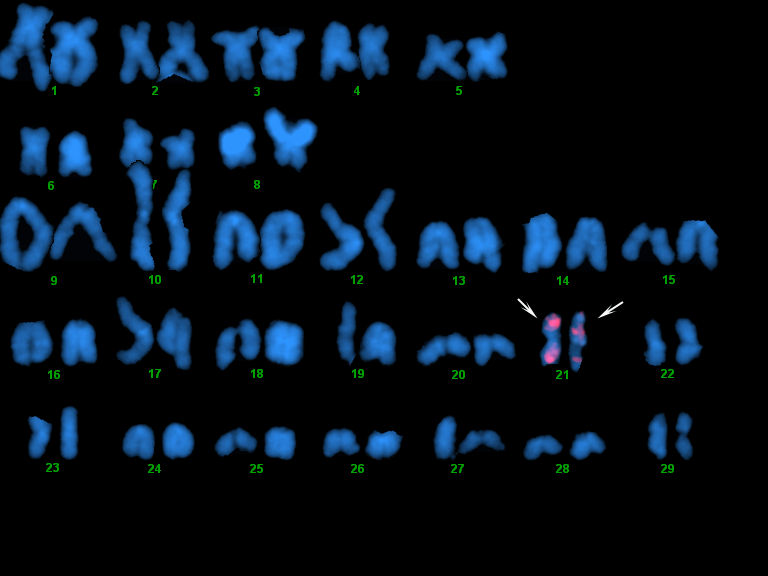

Supplement: Additional file 9 — Fluorescent in situ hybridization of dax1-containing BAC. [file 1471-2164-12-31-S9.TIFF]

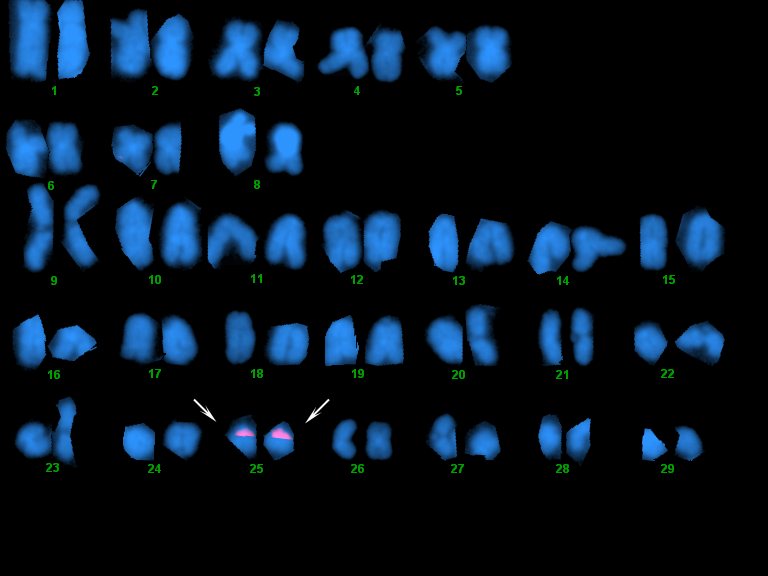

Supplement: Additional file 10 — Fluorescent in situ hybridization of dax2-containing BAC. [file 1471-2164-12-31-S10.TIFF]

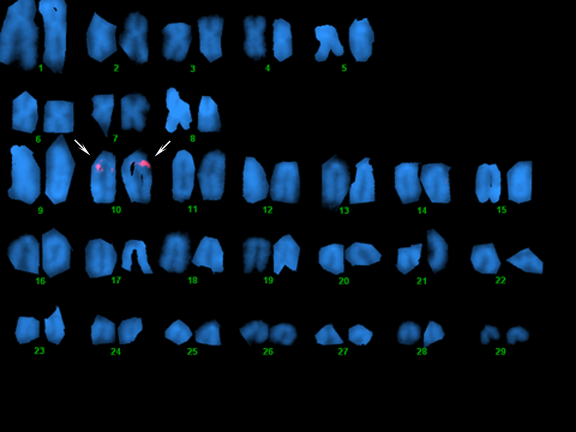

Supplement: Additional file 11 — Fluorescent in situ hybridization of mis-containing BAC. [file 1471-2164-12-31-S11.TIFF]
